# From visceral to visible: A systematic review and meta-analysis of post-kala-azar dermal leishmaniasis incidence, risk factors, and treatment outcomes in Eastern Africa

**DOI:** 10.1371/journal.pntd.0014666

**Published:** 2026-08-18

**Authors:** Eyob Girma Abera, Surafel Worku Megersa, Kedir Negesso Tukeni, Ermias Habte Gebremichael

**Affiliations:** 1 Department of Public Health, Jimma University, Jimma, Oromia, Ethiopia; 2 Jimma University Clinical Trial Unit, Jimma, Oromia, Ethiopia; 3 Department of Psychiatry, St. Paul Hospital Millennium Medical Collage, Addis Ababa, Ethiopia; 4 Department of Internal Medicine, Jimma University, Jimma, Oromia, Ethiopia; US Food and Drug Administration, UNITED STATES OF AMERICA

## Abstract

**Background:**

Post-kala-azar dermal leishmaniasis (PKDL) is a neglected complication of visceral leishmaniasis (VL) treatment with significant public health implications, particularly in Eastern Africa where it can sustain interepidemic transmission. Despite its importance, no comprehensive synthesis of PKDL burden, risk factors, and treatment outcomes specific to the region exists.

**Methods:**

This systematic review and meta-analysis followed PRISMA 2020 guidelines and JBI methodology. A comprehensive search was conducted across PubMed/MEDLINE, Scopus, AJOL, and the Cochrane Library from database inception through May 12, 2026. Studies reporting PKDL incidence, risk factors, or treatment outcomes among VL patients in Eastern Africa were eligible. Random-effects meta-analysis was used to pool cumulative incidence estimates, and heterogeneity was assessed using the I^2^ statistic. For risk factor and treatment outcome studies, a narrative synthesis were performed. Meta-analyses were performed using the meta package in R version 4.5.2 (2025-10-31).

**Results:**

Eleven studies encompassing 4,699 patients from Sudan, Ethiopia, Kenya, and Uganda were included. The pooled cumulative PKDL incidence was 18.2% (95% CI: 4.7%–49.7%), with substantial heterogeneity (I^2^ = 98.1%). Follow-up duration for PKDL assessment varied across studies, ranging from 6 to 24 months. Significant geographic and temporal variation was observed. Sudan reported the highest pooled incidence (56.7%) in studies conducted during the SSG-monotherapy era (1994–2000), compared to Ethiopia (4.6%, 2021) and multi-country cohorts (8.2%, 2022), with more recent supervised treatment data from Sudan and Kenya (2024) showing substantially lower incidence. A temporal decline was noted, from 56.7% pre-2010 to 6.4% post-2010, coinciding with the transition away from sodium stibogluconate monotherapy. Key risk factors included VL treatment regimen, younger age, geographic location, and HIV co-infection. The miltefosine plus paromomycin (MF + PM) combination achieved a clinical cure rate of 98.2% in a Phase II trial, outperforming liposomal amphotericin B combinations.

**Conclusion:**

PKDL remains a significant post-treatment complication in Eastern Africa, with treatment regimen as the most critical modifiable risk factor. MF + PM shows promise as a preferred first-line PKDL therapy. Standardized surveillance and multicountry prospective studies are urgently needed to support regional elimination goals.

**Registration number:**

CRD420261411634.

## Author summary

Post-kala-azar dermal leishmaniasis (PKDL) is a skin condition that can develop in people after they are treated for visceral leishmaniasis (VL, also known as kala-azar), a potentially fatal parasitic infection spread by sandfly bites. People with PKDL develop skin lesions and can also help spread the parasite to new sandflies, making the condition important for disease control, not just individual health. We reviewed all available published studies on PKDL in East Africa to understand how common it is, who is most at risk, and how well it can be treated. We found that PKDL risk varies widely across the region, with Sudan reporting the highest rates and young children being especially vulnerable. The type of drug treatment used for the original VL infection made a major difference: PKDL became less common as an older, single-drug treatment (SSG monotherapy) was phased out in favor of combination therapies. For treating PKDL itself, a combination of two drugs (miltefosine and paromomycin) showed particularly promising results. These findings can help guide treatment choices and target prevention efforts where they are needed most.

## Introduction

Visceral leishmaniasis (VL), also known as kala-azar, is a systemic parasitic disease caused predominantly by Leishmania donovani, with regional strain variation across Eastern Africa, and transmitted by female phlebotomine sandflies. The disease is fatal if untreated, with an estimated 50,000–90,000 new cases annually worldwide [1]. Eastern Africa, particularly Sudan, Ethiopia, Kenya, and Uganda, bears a substantial proportion of the global VL burden, with recurrent outbreaks and high endemicity in rural and semi-arid regions [2].

Post-kala-azar dermal leishmaniasis (PKDL) is a unique complication occurring after apparently successful treatment of VL. It is characterized by a spectrum of skin lesions, including macules, papules, and nodules, typically appearing on the face, trunk, and extremities [3]. The condition is of particular public health importance because PKDL patients may serve as reservoirs for Leishmania transmission, potentially sustaining interepidemic VL outbreaks [4,5].

The epidemiology of PKDL differs markedly between Eastern Africa and the Indian subcontinent. In Eastern Africa, PKDL occurs predominantly in young children within months of VL treatment, reflecting a developing immune response to Leishmania [6]. In contrast, South Asian PKDL typically affects adolescents and adults years after VL cure, suggesting possible immune downregulation [7]. These regional differences underscore the need for region-specific evidence to guide clinical management and control strategies.

Several risk factors for PKDL have been proposed, including young age, male sex, inadequate VL treatment, specific drug regimens, HIV co-infection, and host genetic susceptibility [6,8,9]. However, the evidence base remains fragmented, with inconsistent findings across studies and settings. The role of treatment regimen selection, particularly the shift from sodium stibogluconate (SSG) monotherapy to combination regimens such as SSG plus paromomycin (SSG + PM) or miltefosine plus paromomycin (MF + PM), has emerged as a critical modifiable determinant of PKDL risk [8].

Treatment options for PKDL have historically relied on prolonged courses of parenteral SSG, which is associated with significant toxicity and poor adherence [10]. Recent advances, including short-course combination therapies and immunotherapeutic approaches, offer promise but require systematic evaluation [11,12]. Despite ongoing elimination efforts, PKDL remains a neglected complication, with no standardized surveillance protocols or treatment guidelines specifically tailored to Eastern African settings [13].

Given the evolving treatment landscape and the critical role of PKDL in VL transmission dynamics, a comprehensive synthesis of current evidence on PKDL incidence, risk factors, and treatment outcomes in Eastern Africa is urgently needed. This systematic review and meta-analysis aims to address this gap by pooling available data to inform clinical practice, guide elimination strategies, and identify priorities for future research.

## Methodology

The protocol was registered in the International Prospective Register of Systematic Reviews (PROSPERO) under registration number CRD420261411634. This systematic review was conducted following the Joanna Briggs Institute (JBI) methodology [14], in line with the updated Preferred Reporting Items for Systematic Reviews and Meta-Analyses (PRISMA 2020) guidelines [15] (S1 File).

### Eligibility criteria

Studies were included if they involved patients with VL or PKDL in East African countries, including Ethiopia, Sudan, Kenya, Uganda, and neighboring endemic settings. Eligible study designs varied by research objective: cohort studies (both prospective and retrospective) were considered for PKDL incidence estimation; cohort and case-control studies were considered for risk factor identification; and cohort studies and clinical trials were considered for treatment outcome assessment. Studies were required to report at least one relevant outcome, including PKDL incidence, risk factors, or treatment outcomes. Only articles published in English were considered for inclusion.

Studies were excluded if they were conducted outside East Africa or did not involve VL or PKDL populations. Case reports, review articles, editorials, conference abstracts without sufficient data, commentaries, and letters were excluded. Animal studies, laboratory-based investigations without clinical outcomes, and studies that did not report relevant PKDL outcomes were also excluded.

### Information sources and search strategy

A comprehensive literature search was conducted in PubMed/MEDLINE, Scopus, African Journals Online (AJOL), and the Cochrane Library from database inception to the final search date. To minimize publication bias, grey literature sources, including Google Scholar, were also searched. The search strategy was developed using a combination of Medical Subject Headings (MeSH) and free-text terms related to PKDL, East Africa, incidence, risk factors, and treatment outcomes. Search terms were combined using Boolean operators (“AND” and “OR”) and adapted for each database. No date or language restrictions were applied, and all eligible studies published from database inception through 12 May 2026 were considered for inclusion. Detailed search strategies for all databases are provided in S2 File.

### Study selection

All identified records were imported into EndNote, and duplicate records were removed. Study selection was conducted independently by two reviewers (EGA and SWM). Titles and abstracts were screened against the predefined eligibility criteria, and full-text articles were retrieved for studies that met the inclusion criteria or where eligibility could not be determined from the abstract alone. Any disagreements during the screening or full-text review process were resolved through discussion and consensus. Where consensus could not be reached, a third reviewer (EHG) adjudicated the decision. The study selection process was documented using a PRISMA 2020 flow diagram, detailing the numbers of records identified, screened, excluded (with reasons), and included in the final review.

### Outcome measurements

The primary outcome was the cumulative incidence of PKDL among patients treated for VL in Eastern Africa. Secondary outcomes included risk factors associated with PKDL development and treatment outcomes among patients diagnosed with PKDL. Data on incidence were extracted and pooled using random-effects meta-analysis.

Risk factors of interest included treatment-related factors (VL treatment regimen and treatment adequacy), demographic characteristics (age and sex), geographic location, and immunological or clinical factors such as human immunodeficiency virus (HIV) co-infection and leishmanin skin test response. Treatment outcomes included clinical cure, treatment response, treatment failure, relapse, and adverse events associated with different PKDL treatment regimens, including combination therapies, monotherapies, and immunotherapeutic approaches.

### Data extraction

A standardized data extraction form was developed in Microsoft Excel and piloted on a subset of included studies. Two reviewers (EGA and SWM) independently extracted data from all eligible studies. Any discrepancies were resolved through discussion and consensus, with a third reviewer (EHG) consulted when agreement could not be reached. Extracted information included study characteristics (author, year, country, study design, and sample size), participant characteristics, PKDL incidence, risk factors, treatment regimens, treatment outcomes, follow-up duration, and key findings relevant to the review objectives (S3 File).

### Risk of bias and quality assessment

Risk of bias and methodological quality of the included studies were independently assessed by two reviewers (EGA and SWM), with disagreements resolved through consultation with a third reviewer (EHG). Observational studies were evaluated using the Newcastle-Ottawa Scale (NOS), focusing on selection of study groups, comparability, and outcome ascertainment, with studies scoring 7–9 stars considered to be of high quality and low risk of bias. Randomized controlled trials were assessed using the Cochrane Risk of Bias 2 (RoB 2) tool, which examined potential bias arising from randomization, deviations from intended interventions, missing outcome data, outcome measurement, and selective reporting (S4 File).

### Data synthesis and statistical analysis

We performed meta-analyses using the meta package in R version 4.5.2 (2025-10-31). For cumulative incidence, we pooled study-specific proportions using a random-effects model with the inverse variance method and logit transformation to stabilize variances. The restricted maximum likelihood (REML) estimator was used for between-study variance (τ²). Random effects were modelled at the individual-study level (study-specific proportions pooled with the REML estimator for τ²). Country was used only as a subgroup moderator in descriptive subgroup analyses, not as a nested random-effects level, since too few studies existed per country to support that. Heterogeneity was assessed using the I^2^ statistic and Cochran’s Q test, with I^2^ > 50% indicating substantial heterogeneity [16].

Subgroup analyses were conducted to explore sources of heterogeneity by country (Sudan, Ethiopia, Kenya, multi-country), study design (prospective cohort, retrospective cohort, randomized controlled trial), and temporal era (pre-2010 vs. 2010 onwards). Between-group differences were tested using a mixed-effects meta-regression model. A key limitation of this approach is that age, country, era, and regimen are reported at the aggregate study level, not at the individual patient level, preventing formal multivariable adjustment for these interrelated factors.

Sensitivity analysis was performed using a leave-one-out approach, sequentially omitting each study to assess its influence on the pooled estimate. Publication bias was assessed visually using a contour-enhanced funnel plot, though formal statistical tests were not performed due to the limited number of studies (k < 10), which have insufficient power to reliably detect asymmetry.

For risk factor and treatment outcome studies, we performed a narrative synthesis due to heterogeneity in study designs, exposure definitions, and outcome measures. Effect estimates from individual studies are presented with their 95% confidence intervals (CIs). All statistical tests were two-sided, with p < 0.05 considered statistically significant.

### Certainty of evidence

We assessed the certainty of evidence using the Grading of Recommendations Assessment, Development and Evaluation (GRADE) framework, considering risk of bias, inconsistency, indirectness, imprecision, and publication bias. Certainty was rated as high, moderate, low, or very low for each outcome, with downgrading decisions justified based on the predefined criteria. For the pooled cumulative incidence estimate, certainty was downgraded for serious risk of bias, very serious inconsistency, and serious imprecision. For risk factor and treatment outcome associations, certainty was rated separately by domain based on the strength and consistency of available evidence.

## Results

**Fig 1:**
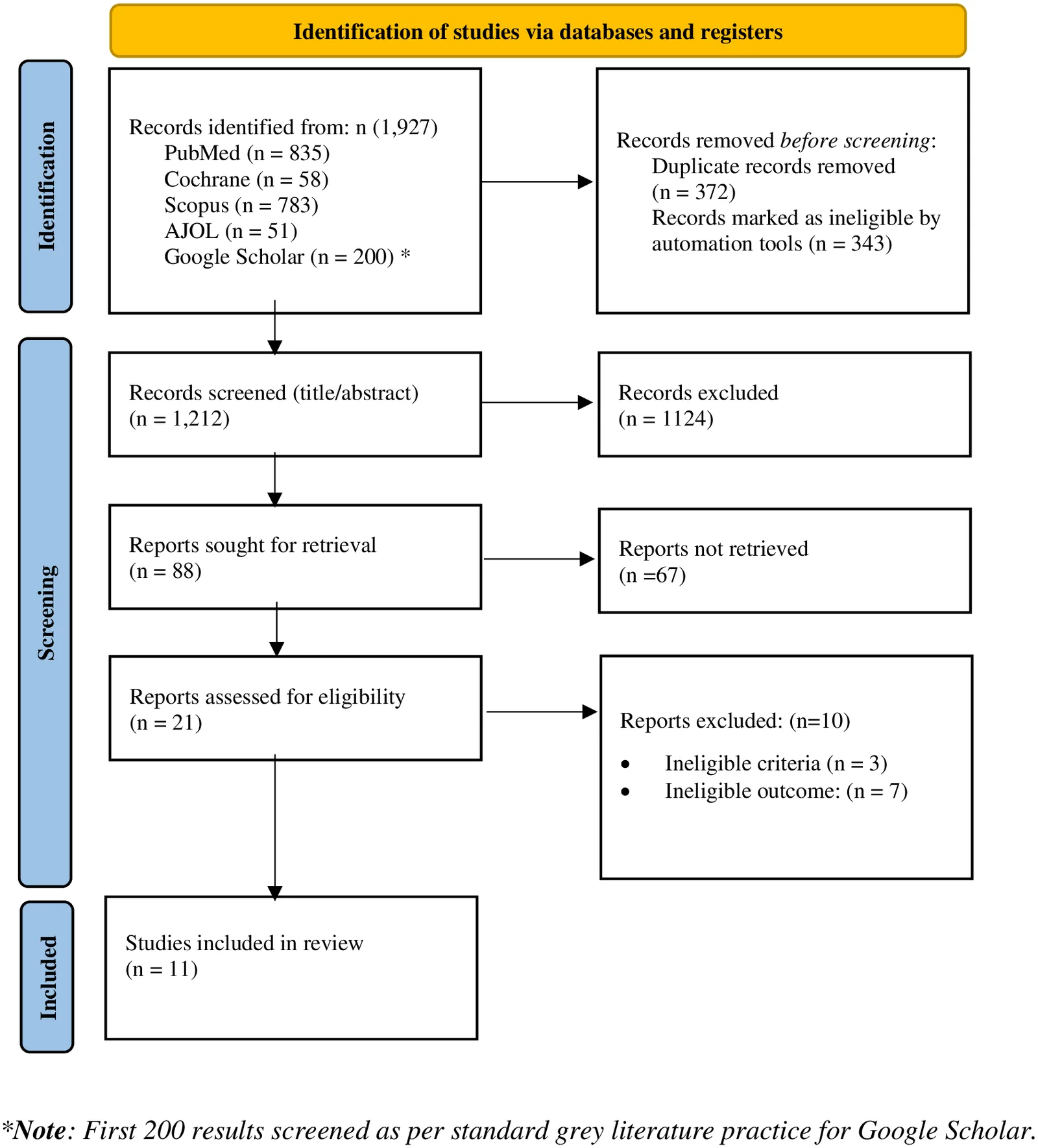
PRISMA flow diagram of search and study selection process.

Out of 1,927 retrieved studies, 11 were eligible and included in the final analysis (Fig 1).

### Characteristics of included studies

The synthesized evidence base comprises 11 distinct studies spanning more than three decades (1992–2024), encompassing an aggregated sample size of 4699 patients across Eastern Africa, primarily concentrated in Sudan, Ethiopia, Kenya, and Uganda. Methodologically, the literature presents a diverse operational profile: five cohorts focused primarily on primary quantitative tracking to report prevalence or cumulative incidence [8,17–20]. Follow-up duration for PKDL assessment varied across these five incidence studies: Zijlstra et al. (1994, 2000) followed patients for up to 12 months or longer; Musa et al. (2022) and Tamiru et al. (2021) had 6-month follow-up; and Kennedy et al. (2024) had variable follow-up ranging from 10 to 24 months (mean 17 months). Sample sizes varied drastically by study design and clinical scope, ranging from intensive localized therapeutic pilot trials or case series evaluating fewer than 20 patients to massive, real-world prospective registries monitoring up to 3,126 individuals under active surveillance (Table 1 and S3 File).

**Table 1:** Baseline characteristics and weight distribution of included PKDL studies.

Authors	Year	Country	Study Design	Sample Size
Zijlstra et al. [17]	1994	Sudan	Prospective cohort	85
Zijlstra et al. [18]	2000	Sudan	Prospective cohort	183
Musa et al. [8]	2022	Ethiopia, Sudan, Kenya, Uganda	RCT	340
Tamiru et al. [19]	2021	Ethiopia	Retrospective cohort	280
Kennedy et al. [20]	2024	Kenya	Retrospective cohort	36
Kimutai et al. [9]	2017	Sudan, Kenya, Ethiopia, Uganda	Prospective cohort	3,126
El-Hassan et al. [21]	1992	Sudan	Retrospective clinical case series	19
Musa et al. [22]	2005	Sudan	Prospective open-label pilot trial	12
Abongomera et al. [10]	2016	Sudan	Retrospective cohort	422
Younis et al. [11]	2023	Sudan	RCT	110
Younis et al. [12]	2024	Sudan	RCT	86

RCTs: Randomized controlled trials.

### Pooled prevalence of PKDL

**Fig 2:**
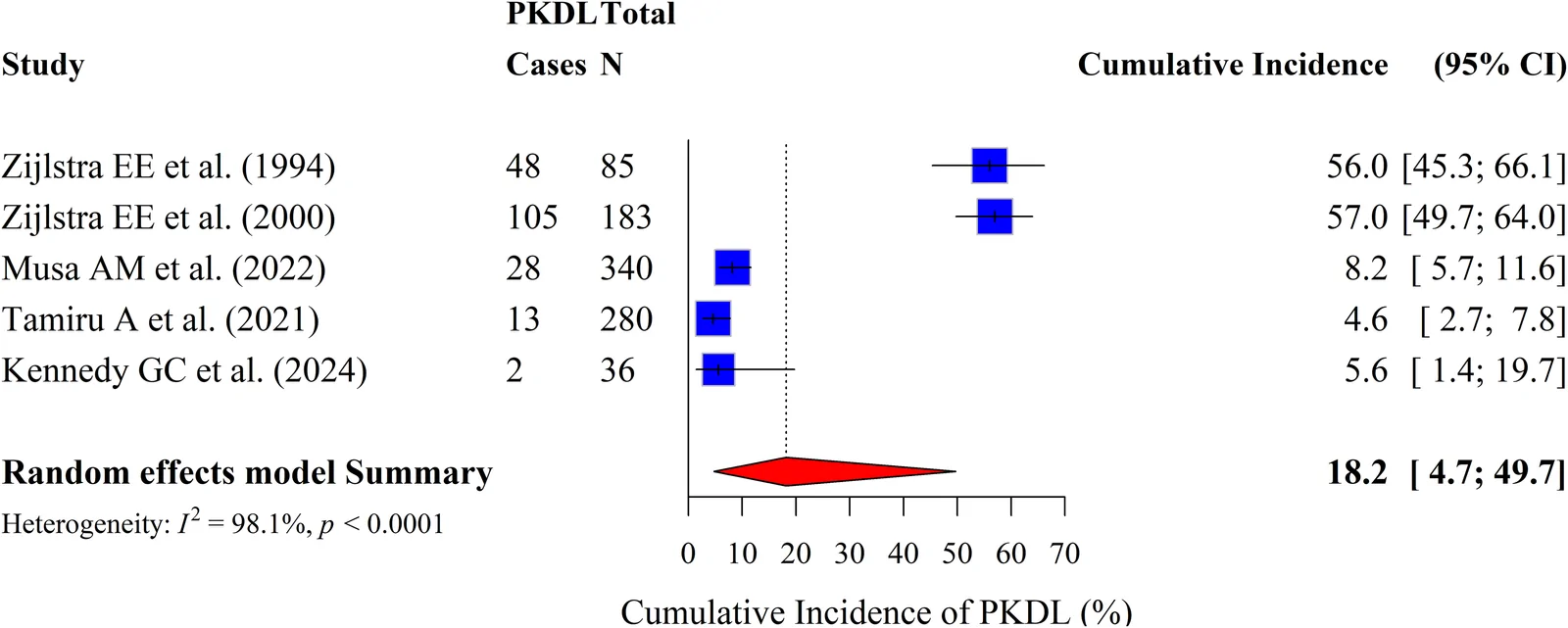
Forest plot of the cumulative incidence of PKDL in Eastern Africa. The summary estimate is derived using a random-effects model (inverse variance method via logit transformation). Individual study proportions are represented by blue squares, with sizes proportional to their relative weight in the baseline analysis. The random-effects weights are as follows: Zijlstra et al. 1994 (20.59%), Zijlstra et al. 2000 (20.78%), Musa et al. 2022 (20.66%), Tamiru et al. 2021 (20.36%), and Kennedy et al. 2024 (17.61%). Horizontal lines represent 95% CIs.

Among the 924 patients from the five studies contributing cumulative incidence data, the pooled cumulative incidence of PKDL among treated visceral leishmaniasis patients in East Africa was 18.2% (95% CI: 4.7%–49.7%) based on a random-effects meta-analysis, with substantial between-study heterogeneity (I^2^ = 98.1%) and a follow-up duration ranging from 6 to 24 months across studies (Fig 2).

### Subgroup and heterogeneity analysis

**Fig 3:**
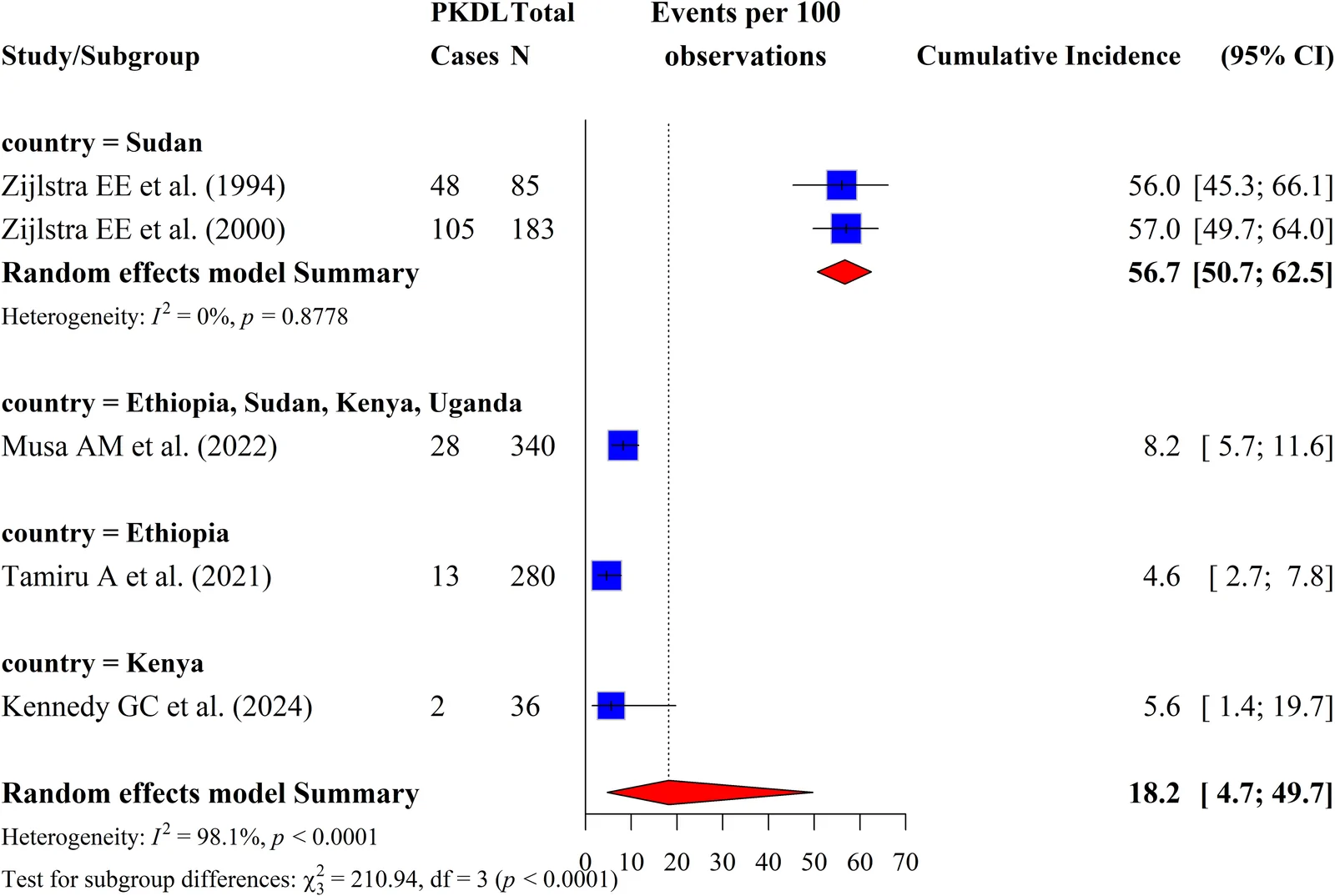
Subgroup analysis of the cumulative incidence of PKDL stratified by country. Individual study estimates and corresponding 95% CIs are grouped by geographic setting (Sudan, Ethiopia, Kenya, and multi-country cohorts). Subgroup summary diamonds reflect random-effects pooling within each country stratum.

Although studies for subgroup analysis are few and highly individualized by country, Sudan emerged as the clear high-risk epicenter with an exceptional pooled PKDL cumulative incidence of 56.7%. In stark contrast, neighboring East African nations and regional multicentre trials demonstrated much lower, single-digit risks ranging from 4.6% in Ethiopia to 8.2% regionally. This profound geographic divergence was statistically confirmed by a highly significant test for between-group differences (χ² = 210.94, df = 3, p < 0.0001), establishing country location as a primary driver of the overall heterogeneity (Fig 3).

**Fig 4:**
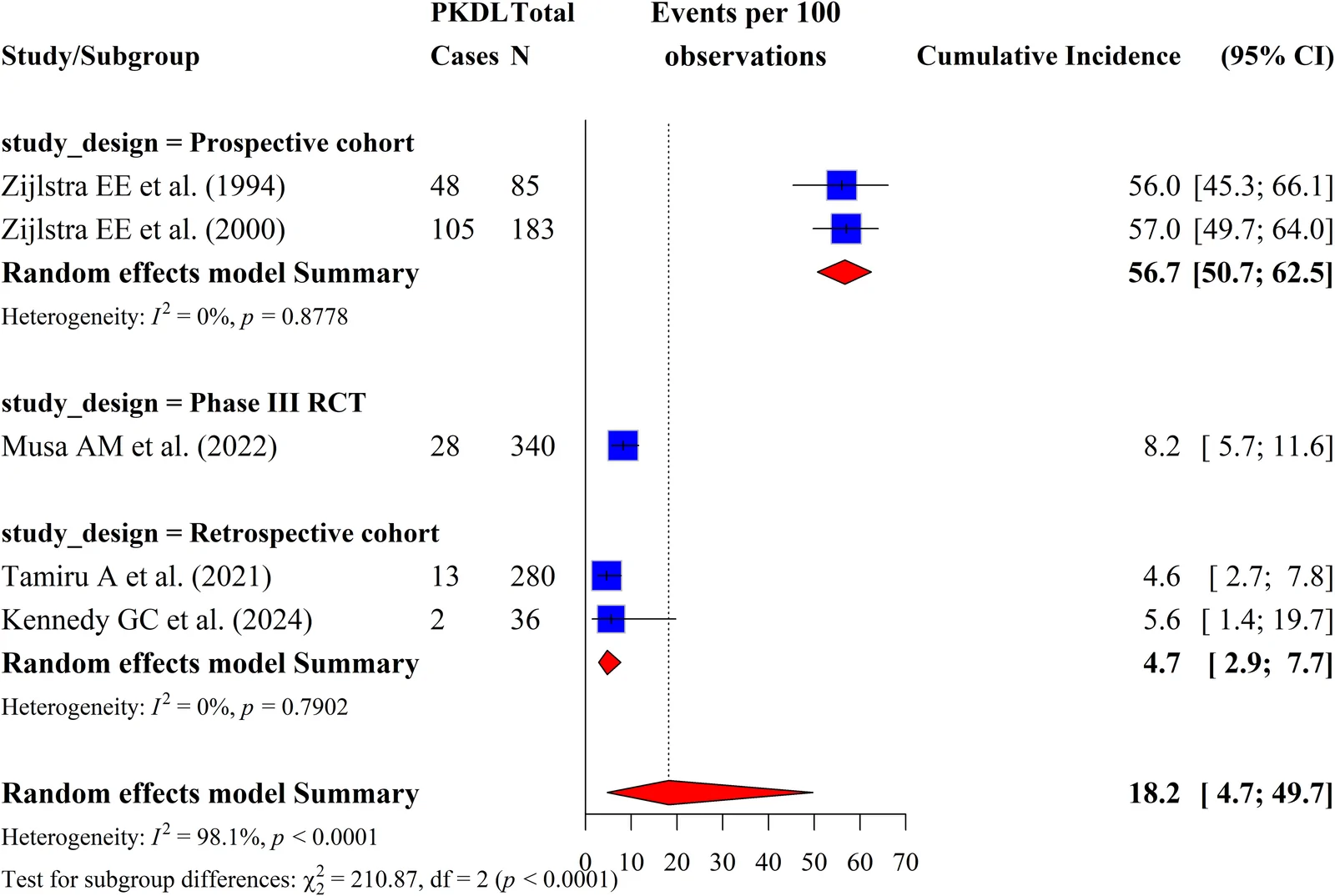
Subgroup analysis of the cumulative incidence of PKDL stratified by study design. Included cohorts are partitioned into prospective cohorts, retrospective cohorts, and RCTs. The red diamonds display the pooled stratum-specific cumulative incidence. Between-study variance metrics and group-specific statistical weights are detailed in the lower margins.

Study design also significantly influenced observed incidence (p < 0.001). Prospective cohort studies showed the highest pooled incidence (56.7%, 95% CI: 50.7%–62.5%), followed by randomized controlled trials (8.2%, 95% CI: 5.8%–11.7%) and retrospective cohort studies (4.7%, 95% CI: 2.9%–7.7%) (Fig 4).

**Fig 5:**
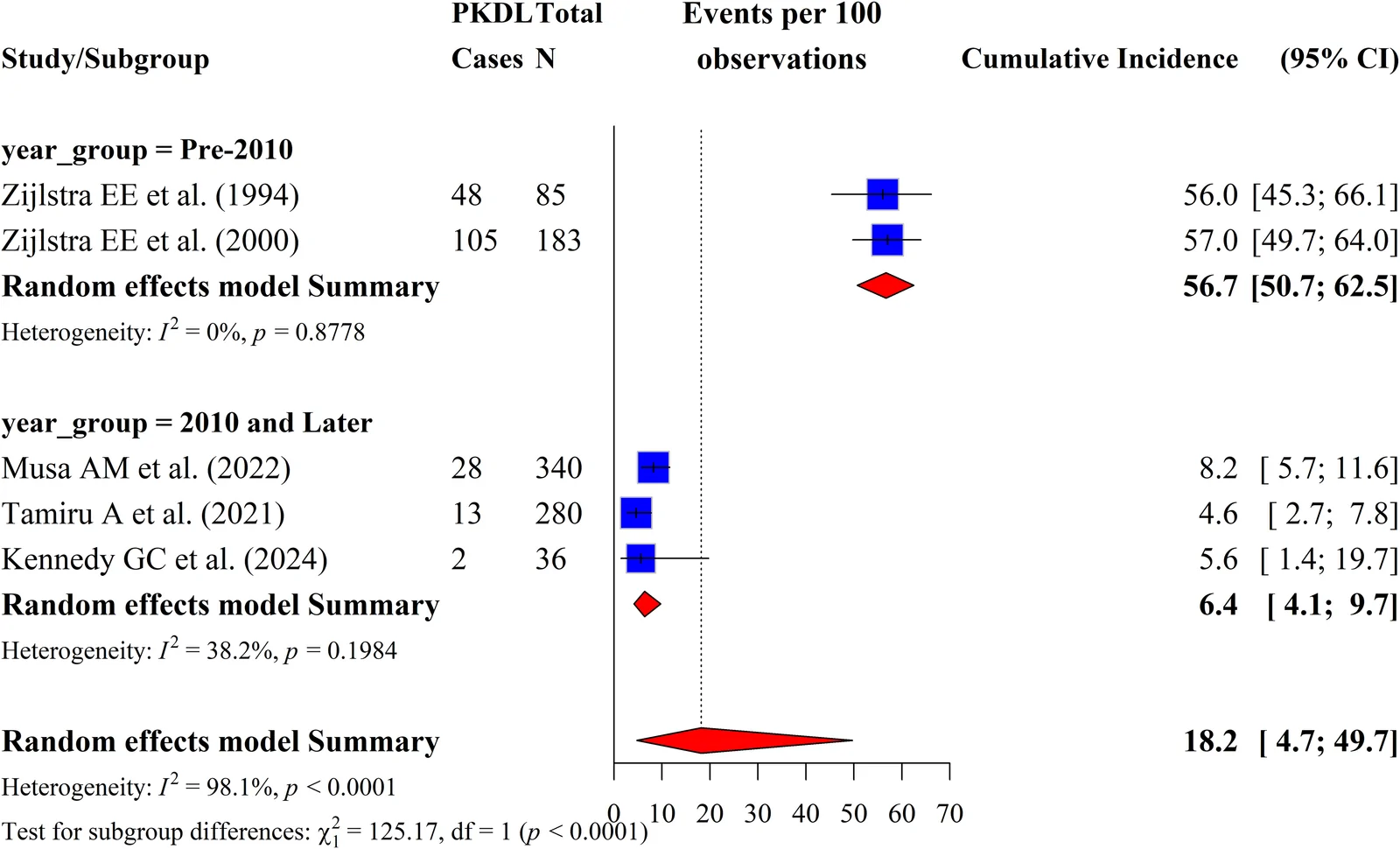
Subgroup analysis of the cumulative incidence of PKDL stratified by operational era. Studies are dichotomized into historical cohorts published prior to 2010 (“pre-2010”) and modern cohorts published from 2010 onward (“2010 and Later”) to evaluate temporal shifts in incidence patterns across Eastern Africa. Strata summaries are generated using a random-effects model.

A clear temporal decline was also observed (p < 0.001). Studies conducted before 2010 reported a pooled incidence of 56.7% (95% CI: 50.7%–62.5%), whereas studies from 2010 onwards showed a substantially lower incidence of 6.4% (95% CI: 4.1%–9.7%) (Fig 5).

### Sensitivity analysis

A leave-one-out sensitivity analysis showed that the pooled PKDL cumulative incidence was robust to the exclusion of individual studies. The pooled estimate ranged from 12.3% to 24.9% across iterations, while heterogeneity remained consistently high (I^2^: 97.4%–98.5%), indicating that no single study substantially influenced the overall findings (S1 Fig).

### Publication bias

Visual inspection of the contour-enhanced funnel plot suggested some degree of asymmetry, with study estimates distributed unevenly around the pooled effect axis. However, interpretation remains strictly limited given that only five studies were included, falling below the recommended threshold of 10 studies where funnel plots and formal regression diagnostics have sufficient power to reliably detect publication bias. Consequently, the observed asymmetry likely reflects the substantial baseline between-study heterogeneity (I^2^ = 98.1%) across differing operational eras rather than true publication bias alone (S2 Fig).

### Risk Factors of PKDL

#### 1. Therapeutic and modifiable factors.

Across the literature, primary VL treatment choice and clinical adequacy emerged as the most critical modifiable determinants governing subsequent PKDL onset. The most robust experimental evidence comes from the AfriKADIA Consortium’s Phase III randomized controlled trial sub-analysis (2017–2021) spanning Ethiopia and Sudan (n = 340 patients within PKDL-relevant arms), which demonstrated that the specific drug combination utilized influences the probability and timing of PKDL development. Patients randomized to the standard SSG + PM regimen exhibited a vastly higher risk of developing dermal complications (13.5%) compared to those treated with the oral and injectable alternative of MF + PM, where the incidence dropped to 2.9% [8].

This is structurally complemented by programmatic data from a massive prospective pharmacovigilance rollout (2010–2014) monitoring the routine field deployment of first-line SSG + PM across Sudan, Kenya, Ethiopia, and Uganda (n = 3,126) [9]. In this real-world setting, which represents the largest aggregated VL dataset in East Africa, disease progression was heavily influenced by baseline clinical status, noting distinct risk variances between individuals presenting with primary VL versus those experiencing a clinical relapse. Furthermore, detailed longitudinal cohort tracking in hyper-endemic Sudanese villages (n = 85 followed ≥6 months in the 1994 cohort and n = 183 followed for incidence in the 2000 cohort) underscored that the precision of initial therapy is paramount; sub-standard or inadequate primary dosing of antimonials significantly accelerated the subsequent cumulative incidence of dermal lesions [17,18].

#### 2. Host demographics (age and sex).

Non-modifiable host demographics, particularly age at primary infection, exert profound biological control over cutaneous parasite replication, whereas host biological sex exhibits less consistent associations. Longitudinal village cohort data from Sudan demonstrated a stark, age-dependent susceptibility profile, revealing that infants and toddlers aged 0–3 years experience a massive escalation in absolute risk for PKDL (78–81%) compared to older cohorts followed during identical time windows [17,18]. However, among patients who develop PKDL, severe disease is more frequent in younger age groups (p < 0.001) [18]. Conversely, data from the 3,126-patient prospective pharmacovigilance registry indicated that patients older than 50 years had a lower initial VL cure rate (81.4%) compared to the overall cure rate of 95.1%, identifying older age as a risk factor for poorer VL treatment outcomes [9].

In rural, highly endemic settings such as the Tiaty sub-counties of Kenya, retrospective and cross-sectional tracking (n = 36 follow-up cohorts combined with 248 hospital records) identified female sex as significantly associated with PKDL (p = 0.04), contrasting with the male predominance observed in Sudan and Ethiopia [20]. The same study validated age as a primary risk separator for VL relapse, though not specifically for PKDL.

#### 3. Spatial and geographical distribution.

The burden of PKDL is highly localized, dictated by both macro-regional variations and micro-geographical, village-level vector dynamics. At a macro-epidemiological level, multi-country clinical evidence shows distinct geographical clustering. The pre-specified sub-group analysis from the AfriKADIA randomized trial showed that individuals managed within the hyper-endemic belts of Sudan and Ethiopia faced markedly higher PKDL risks compared to those treated in Kenya and Uganda, where zero PKDL cases were observed regardless of treatment regimen [8].

This geographical divide is reflected in large programmatic registries, where the vast majority of the regional burden remains highly concentrated in Sudan, comprising 62.7% of the 3,126-patient dataset, followed by Kenya (20.9%), Ethiopia (10.3%), and Uganda (6.1%). These numbers directly mirror varying regional vector densities and underlying transmission intensities across the continent [9]. On a localized scale, recent field data from rural Kenya highlighted significant variation in combined VL/PKDL follow-up outcomes between adjacent administrative sectors, such as Tiaty East versus Tiaty West (p = 0.03) [20].

#### 4. Immunological and co-morbid determinants.

The intersection of host immune system integrity, systematic co-infections, and specific biomarkers functions as a critical biological gatekeeper for post-treatment parasite clearance. Active systemic immunosuppression represents a primary driver of aggressive dermal progression. Large-scale pharmacovigilance data proved that baseline HIV co-infection at primary VL diagnosis significantly modifies cure of the primary VL episode itself; co-infected patients had an initial VL cure rate of only 56% compared to 95.1% overall. However, crude analytical models are heavily confounded by high early mortality and treatment failure among co-infected patients [9].

In highly remote cohorts, the clinical management of immune-compromised individuals remains critical, meaning that concurrent antiretroviral therapy (ART) use must be factored into long-term dermatological surveillance [20]. Furthermore, early longitudinal field studies highlighted that delayed systemic immune responses, specifically measured by delayed or absent leishmanin skin test (LST) conversion, directly correlate with failure to achieve long-term cutaneous parasite containment. Among PKDL patients, there was an inverse correlation between LST conversion and disease severity (p < 0.01) [18].

### Treatment outcome of PKDL

The following section describes outcomes of therapies specifically directed at treating PKDL once it has developed, as distinct from VL treatment regimens that influence the risk of subsequent PKDL.

#### 1. Short-course combination regimens.

Recent therapeutic research in Sudan has focused on evaluating short-course, low-toxicity combination therapies. A Phase II, open-label, parallel-arm, non-comparative randomized controlled trial (2018–2021) conducted at the El Hassan Centre in Doka, Sudan, evaluated 110 randomized patients (104 completed), who were primarily pediatric (median age: 9 years; 87% aged ≤12 years) [11]. The study demonstrated remarkable efficacy for a completely oral or injectable short-course combination of PM/MF, achieving a clinical cure rate of 98.2% (54/55; 95% CI: 90.3–100). The second arm evaluating Liposomal Amphotericin B combined with Miltefosine (LAmB/MF) yielded a lower cure rate of 80.0% (44/55; 95% CI: 70.2–91.9), positioning PM/MF as a highly attractive candidate for pediatric PKDL management.

#### 2. Programmatic outcomes and refractory disease.

Real-world field evidence comes from a large retrospective cohort analysis of Médecins Sans Frontières (MSF) programme data collected over 6 years (2002–2008) in Lankien, South Sudan [10]. The study analyzed 422 patients with severe-grade PKDL. Among those treated with Sodium Stibogluconate monotherapy (SSG; n = 343), the programmatic cure rate was 90%. The addition of Paromomycin (SSG + PM) in 79 severe cases increased the cure rate to 97%, confirming that combination therapy provides superior outcomes even under field conditions.

Managing patients who fail first-line therapy presents a distinct challenge. A prospective, open-label, single-arm pilot trial in Khartoum evaluated a salvage protocol for 12 patients with persistent, severe PKDL (≥6 months duration) who were refractory to standard SSG therapy [22]. By combining SSG with Leishvacin (an immunotherapy vaccine), the study successfully treated 83.3% (10/12) of these persistent cases, suggesting a potential role for immune modulation in refractory disease.

#### 3. Early experience and immunotherapeutic trials.

Early retrospective clinical series established the baseline for PKDL treatment. A study of 19 PKDL patients in Sudan found that intravenous SSG (20 mg/kg for 30 days) achieved a reasonably good response, though some patients required repeated or prolonged treatment [21]. In contrast, oral ketoconazole (10 mg/kg daily for 4 weeks) demonstrated no therapeutic effect, establishing the requirement for dedicated leishmanicidal compounds.

More recently, a Phase IIb randomized, double-blind, placebo-controlled trial (2019–2022) conducted at the University of Khartoum evaluated the ChAd63-KH therapeutic vaccine in 86 patients (75 completed; 87%) aged 12–50 years with chronic PKDL lasting at least 6 months [12]. The clinical cure rate was 15% (6/40) in the vaccine arm compared to 11% (4/35) in the placebo arm, a difference that was not statistically significant (p = 0.742). This high-quality negative trial demonstrated no efficacy for this vaccine candidate, confirming that chemical leishmanicidal combinations remain the standard of care.

### Risk of bias assessment

Overall, the included randomized controlled trials (n = 3) demonstrated low risk of bias, while the non-randomized studies (n = 8) showed variable quality, with NOS scores ranging from 4 to 8 stars. Three studies were rated as good quality [9,17,18], three as fair [10,20,22], and two as poor [19,21], largely due to retrospective design, small sample sizes, or high loss to follow-up (S4 File).

### Certainty of evidence

Using the GRADE framework, the certainty of evidence for the pooled cumulative incidence of PKDL (18.2%) was very low, primarily due to substantial heterogeneity (I^2^ = 98.1%) and wide confidence intervals. For risk factor associations, the certainty was moderate for the effect of VL treatment regimen (SSG + PM vs. MF + PM) based on evidence from randomized controlled trials. The certainty was low for young age and geographic variation, and very low for female sex, HIV co-infection, and biomarker associations due to risk of bias, inconsistency, or imprecision. These findings suggest that while treatment-related risk factors are supported by moderate-certainty evidence, other associations require further investigation.

## Discussion

This systematic review and meta-analysis synthesized over three decades of evidence (1992–2024) from 11 studies encompassing 4,699 patients across four Eastern African nations to characterize the epidemiology, risk factors, and treatment outcomes of PKDL. The pooled cumulative incidence of PKDL among treated VL patients in Eastern Africa was estimated at 18.2% (95% CI: 4.7%–49.7%), accompanied by substantial between-study heterogeneity (I^2^ = 98.1%). Our synthesis identifies primary VL treatment regimen, patient age, geographic locale, and immune status as the primary determinants of PKDL risk, each carrying significant implications for clinical management, surveillance, and regional elimination programs.

The pooled PKDL cumulative incidence of 18.2% (95% CI: 4.7%–49.7%) derived in this review occupies a central position within the upper range of estimates from global endemic settings. This central estimate aligns with the broadly reported global range of 2.5%–20% for PKDL following VL treatment [5,23] and confirms Eastern Africa, as a high-burden epicenter. However, subgroup analysis demonstrated that this aggregate figure masks profound geographic stratification: Sudan alone accounted for a pooled incidence of 56.7%, consistent with long-standing regional estimates of 50%–62% reported in clinical series and consensus reviews [8,24]. In contrast, reported PKDL frequencies in East Africa vary by setting, with historical Sudanese cohorts showing the highest burden and more recent studies suggesting lower rates in some areas, underscoring the influence of ecological, parasitic, host, and treatment-related factors across the region [6].

The extreme heterogeneity observed (I^2^ = 98.1%) is a substantive and clinically meaningful signal. The between-study variance was demonstrably attributable to a cluster of interrelated study-level factors, namely country, study design, and operational era, rather than random sampling error. These factors are not independent of treatment regimen: regimen use is strongly patterned by country and calendar period in this literature, so country, design, and era are best understood as markers of which regimen was in use rather than independent causal drivers. This high level of variance is consistent with published meta-analyses of other neglected tropical disease outcomes in low-resource settings, where operational and ecological diversity generates unavoidable baseline variance [25]. Nevertheless, the robustness of the aggregate estimate across leave-one-out sensitivity iterations (range: 12.3%–24.9%) supports its validity as a stable benchmark for regional policy planning.

The included studies did not report parasite genotyping or vector species data. The wider literature describes different Leishmania donovani strains and different dominant sandfly vectors in Sudan and Northern Ethiopia (Phlebotomus orientalis) compared to Southern Ethiopia and Kenya (P. martini) [26]; this biological heterogeneity is a plausible contributor to the geographic variation in PKDL incidence reported here, but could not be tested with the available aggregate data. Whether the parasite subtype and vector pattern recently reported in the Kenyan outbreak represent a genuinely new combination or a previously undetected one remains unresolved and warrants targeted genotyping and entomological study [27].

Host genetic susceptibility to PKDL is also known to vary both within and between countries, with reported differences in PKDL rates between Sudanese villages sharing similar ecology and certain ethnic groups disproportionately affected [6,28]. None of the included studies reported host genotyping or ethnicity-stratified incidence, so this source of heterogeneity could not be quantified.

HIV co-infection among VL patients is common in Ethiopia but only rarely reported in Sudanese cohorts [29,30]. Because our HIV-related findings are drawn predominantly from the multi-country pharmacovigilance registry, they should not be assumed to generalize uniformly across countries with very different underlying HIV-VL overlap.

PKDL in Sudan occurs predominantly 0–13 months after VL treatment [6], whereas the randomized controlled trials included in this review followed patients for only 6 months. Incidence estimates derived from these trials likely underestimate true PKDL incidence and should be interpreted as minimum estimates.

A substantial part of the very high early Sudanese PKDL estimates has been attributed to unsupervised, sub-standard SSG administration, including diluted or adulterated drug given at low, intermittent doses by unqualified health workers, rather than to the choice of SSG as a regimen per se. More recent Sudanese data from supervised, quality-controlled treatment report substantially lower incidence (1–2%), supporting treatment adequacy, not merely drug choice, as a key modifiable driver [31].

Unlike the Indian subcontinent, where widespread clinical SSG resistance was a principal driver of the shift away from antimonial monotherapy, East African L. donovani has not shown comparable levels of documented SSG resistance [32]. The regional decline in SSG use appears to be driven predominantly by toxicity, adherence, and treatment-supervision concerns rather than parasitological resistance.

Sex differences in PKDL risk were inconsistent across included studies. The direction of this association varied by setting, with male predominance reported in Sudan and Ethiopia but female association in the Kenyan data [6]. None of the included studies tested explanatory mechanisms directly. Commonly proposed but unverified explanations include differential outdoor or occupational sandfly exposure and differential health-care-seeking behavior by sex. We flag these as hypotheses for future studies rather than established findings.

Finally, PKDL is increasingly understood to reflect persistence of viable Leishmania parasites in the skin despite systemic clearance of VL, potentially reflecting sub-therapeutic drug penetration into dermal tissue [33]. None of the included studies measured tissue drug levels or dermal parasite persistence directly; pharmacokinetic and pharmacodynamic studies of drug penetration into skin represent a priority for future research linking inadequate primary treatment to subsequent PKDL.

The profound geographic divergence in PKDL incidence most starkly seen between Sudan (56.7%) and Kenya or multi-country cohorts (8.2%), stems from distinct biological and programmatic factors. Sudan represents the most intense PKDL-endemic environment in East Africa due to a high primary VL burden, decades of reliance on prolonged SSG monotherapy, and a hyper-endemic Phlebotomus orientalis sandfly ecosystem in rural transmission zones [21]. It is important to note that almost all Sudanese studies included in this review were conducted during the SSG-monotherapy era, meaning country and treatment regimen are near-perfectly collinear at the study level. Individual-patient data needed to statistically separate these effects were not reported in the included studies. This 56.7% estimate should therefore be interpreted as characterizing the historical Sudanese SSG-monotherapy era specifically, rather than as an independent country-level effect. This interpretation is supported by supervised, routine-conditions treatment data from Sudan showing substantially improved outcomes under SSG + PM combination therapy, consistent with treatment adequacy, not merely country, being the operative factor [31].

In contrast, the low or absent PKDL incidence reported from Kenya and Uganda, including zero PKDL cases in the AfriKADIA Phase III trial [8], reflects lower transmission intensities and the earlier integration of alternative combinations. These geographic findings are consistent with previous evidence that PKDL burden varies substantially across endemic settings because of differences in vector ecology, parasite strains, host factors, and treatment history. The observed decline in PKDL incidence may also reflect changes in visceral leishmaniasis treatment, as historical data from Bihar suggested that increased amphotericin B use coincided with fewer PKDL cases [6].

The temporal analysis revealed a striking and highly significant decline in PKDL incidence from 56.7% in studies conducted before 2010 to 6.4% in studies from 2010 onward (p < 0.001). This trajectory mirrors the phased transition in Eastern Africa from SSG monotherapy toward SSG + PM combination regimens and miltefosine-based therapies. The observation that the decline in PKDL incidence coincided with the reduction in antimonial use is corroborated by historical reports noting that PKDL burdens declined concomitantly with the expansion of amphotericin B and combination alternatives [34]. This matches trends on the Indian subcontinent where widespread shifts away from SSG first-line therapy reduced PKDL rates, though shifting case-ascertainment methods and improved surveillance infrastructure across eras may also independently contribute to this decline.

The choice of primary VL treatment regimen is the single most critical modifiable determinant of subsequent PKDL risk. The AfriKADIA Phase III randomized controlled trial demonstrated that patients treated with SSG + PM had a 13.5% PKDL incidence compared to only 2.9% among those receiving MF + PM, a nearly five-fold difference [8]. This finding has profound public health implications given that MF + PM was non-inferior to SSG + PM for primary VL cure while removing SSG-associated cardiotoxicity and reducing downstream PKDL complications [35]. Mechanistically, antimonials alter dermal parasite clearance, leading to persistent low-grade infections and unique immunopathological cascades, including cellular IL-10 overexpression and IFNGR1 downregulation [6].

Programmatic pharmacovigilance data from the 3,126-patient registry further reinforced this relationship, showing that disease progression was heavily influenced by baseline clinical status, with relapsing VL patients carrying a heightened risk relative to primary cases [9]. Visceral leishmaniasis relapse is associated with deeper immune dysregulation and greater cumulative parasite burdens, predisposing the host to dermal dissemination [10,31]. Collectively, these findings reinforce the urgent need to implement MF + PM or alternative non-antimonial combinations as first-line VL therapy across Eastern African health systems to concurrently reduce primary morbidity and interrupt downstream PKDL development.

Young age at primary VL infection emerged as the most consistent non-modifiable risk factor for PKDL in Eastern Africa. Sudanese longitudinal cohorts documented that infants and toddlers aged 0–3 years had a cumulative PKDL risk of 78%–81% during the period when unsupervised SSG monotherapy was the standard treatment, with substantially lower risk in older children and adults from the same cohorts, confirming that this age gradient was observed within, not across, treatment eras [17,18]. This susceptibility profile starkly contrasts with South Asia, where PKDL predominantly affects adolescents and adults after an extended latency period, reflecting fundamentally different immunopathological trajectories [6]. In Eastern Africa, the immature immune response in very young children coupled with an undeveloped skin immune microenvironment likely facilitates rapid cutaneous parasite seeding, resulting in the rapid post-treatment PKDL onset (within 1–13 months) characteristic of the region [6].

Conversely, data from the large-scale pharmacovigilance registry indicated that patients aged over 50 years had significantly higher primary cure rates [9], supporting the hypothesis that a mature adaptive immune response effectively suppresses residual cutaneous parasites. With respect to sex, findings were inconsistent: while male predominance is typical in Sudan and Ethiopia, Kenyan data identified female sex as a significant PKDL risk factor (p = 0.04) [20]. These conflicting findings likely reflect location-specific behavioral, environmental, and healthcare-seeking confounders rather than intrinsic biological pathophysiology, as the broader literature indicates a male-to-female ratio of roughly 1.8:1 in Eastern African field settings [6].

Beyond macro-regional stratification, our review uncovered evidence of micro-geographical clustering in VL/PKDL follow-up outcomes, with significant variation between adjacent administrative sectors in Kenya (Tiaty East vs. Tiaty West; p = 0.03) [20]. It is important to note that the source study did not adjust for underlying VL incidence by sub-county, so this finding cannot be interpreted as a PKDL-specific spatial effect independent of VL risk. Such localized spatial heterogeneity is consistent with known determinants of Phlebotomus vector ecology, terrain, soil composition, and domestic proximity to breeding sites, which most plausibly act on primary VL transmission intensity rather than PKDL risk specifically [2]. This finding therefore supports combined VL/PKDL surveillance rather than a PKDL-specific spatial effect. The heavy burden concentration in Sudan (62.7% of the dataset) further confirms that regional investments must be proportionally focused on high-intensity Sudanese transmission zones [9].

HIV co-infection was identified as a major immune-mediated amplifier of PKDL risk and treatment failure, with co-infected patients achieving initial VL cure rates of only 56% compared to 95.1% in HIV-negative individuals [9]. The pathophysiological mechanism is well-established: HIV-mediated depletion of CD4 + T lymphocytes impairs the Th1-polarized immune response and IFN-γ production essential for effective leishmanial killing in both visceral and dermal compartments [36]. This creates a highly permissive environment for persistent cutaneous parasite survival post-VL treatment. Concurrent ART must be factored into long-term surveillance protocols, as immune reconstitution under effective ART can partially restore host capacity for cutaneous lesion resolution.

Immunological biomarkers, specifically the LST, emerged as a strong correlate of PKDL severity. The inverse correlation between LST conversion and disease severity (p < 0.01) [18] supports the interpretation that patients with delayed or absent cellular immune reconstitution following VL treatment fail to contain cutaneous parasites. This aligns with the broader immunopathology literature, where elevated plasma IL-10 levels during primary VL predict subsequent PKDL development by suppressing protective Th1 immunity [6], offering a valuable biomarker target for post-treatment surveillance to identify high-risk individuals.

The most clinically significant therapeutic finding in this review is the 98.2% clinical cure rate achieved by PM/MF in a Phase II trial [11]. This positions PM/MF as the most promising candidate for updated clinical guidelines. This high efficacy was achieved in a predominantly pediatric Sudanese cohort (87% aged ≤12 years), the exact demographic bearing the highest absolute PKDL burden [11]. Operationally, PM/MF requires only 14 days of hospital admission for parenteral paromomycin followed by oral miltefosine completion at home, replacing traditional SSG monotherapy which demands 60–90 days of toxic injectable treatment under close supervision [35]. The WHO PKDL guidelines revision process, currently ongoing with new recommendations for Eastern Africa expected by 2026, is anticipated to incorporate PM/MF as a recommended option given this evidence base [13].

In contrast, the liposomal amphotericin B and miltefosine (LAmB/MF) arm of the same trial achieved a lower cure rate of 80.0% [11], positioning it as a second-line option. The inferior performance of LAmB/MF relative to PM/MF likely reflects poor skin pharmacokinetic profiles, as achieving effective liposomal concentrations in cutaneous tissue remains a major pharmacological challenge [13]. Under real-world conditions, programmatic data from South Sudan confirmed that combination therapy specifically SSG + PM yielded superior outcomes (97% cure) compared to SSG monotherapy (90%) in severe PKDL cases [10]. This 7-percentage-point gain from adding paromomycin translates to a meaningful reduction in persistent cases, retreatment burden, and community parasite transmission reservoirs.

The retrospective evaluation of SSG plus Leishvacin (an immunotherapy adjunct) for refractory PKDL achieved an 83.3% success rate in a small salvage protocol [22]. While the sample size is small, this observation highlights immune modulation as a valuable rescue strategy. The persistence of severe PKDL despite adequate antimonial exposure reflects a dermal immune microenvironment failure rather than simple drug resistance, suggesting that severe cases require therapeutic augmentation rather than intensified leishmanicidal chemotherapy alone.

Conversely, the Phase IIb randomized controlled trial of the ChAd63-KH therapeutic vaccine yielded a clearly negative result, with clinical cure rates of 15% in the vaccine arm versus 11% in the placebo group (p = 0.742) among patients with chronic PKDL [12]. This high-quality null finding indicates that long-standing, chronic PKDL involves progressive dermal fibrosis and established immune tolerance that single-agent immune interventions cannot overcome [6]. Future immunotherapy research must explore combination approaches pairing vaccine candidates with adjunctive leishmanicidal therapies to break this immune tolerance barrier.

Foundational data established that while intravenous SSG (20 mg/kg for 30 days) achieved reasonable clinical responses, oral ketoconazole proved completely ineffective [21]. These early data established the leishmanicidal specificity required for effective PKDL therapy and validated SSG as the historical cornerstone of care. However, SSG’s severe toxicity profile, prolonged injectable course, and requirement for intensive inpatient monitoring present massive operational barriers, particularly for pediatric patients [37]. The ongoing transition away from SSG monotherapy toward alternative short-course combinations represents a necessary evolution to relegate toxic antimonials to a minor salvage role.

Beyond its direct clinical burden, PKDL carries critical implications for the regional VL elimination agenda. The Eastern African Strategic Framework for VL Elimination 2023–2030 explicitly identifies the detection and management of PKDL cases as a strategic pillar, reflecting evidence that PKDL patients harbor viable, infectious Leishmania donovani in cutaneous lesions capable of infecting sandfly vectors [13]. Because PKDL patients are typically asymptomatic beyond skin lesions, they frequently delay treatment-seeking, maintaining prolonged parasite skin reservoirs in communities [5,38]. Mathematical modeling demonstrates that these infectious PKDL cases can sustain cryptic transmission cycles that evade standard vector control interventions, threatening long-term elimination efforts [39].

The high incidence of PKDL following standard SSG + PM treatment (20.9%) compared to MF + PM treatment (4.4%) as documented in the AfriKADIA trial carries elimination-level consequences [8]. Replacing SSG + PM with MF + PM as first-line VL therapy across Eastern Africa would directly improve immediate patient safety, minimize the population-level chronic dermal reservoir, and accelerate progress toward the 2030 regional VL elimination targets. This dual benefit provides a compelling, evidence-based argument for the expedited regulatory and programmatic adoption of MF + PM across Eastern African health systems.

### Strengths and limitations

This review provides the first comprehensive synthesis of the incidence, risk factors, and treatment outcomes of PKDL in Eastern Africa. By incorporating evidence from randomized controlled trials, cohort studies, and large programmatic datasets across multiple countries, the review offers a broad overview of PKDL epidemiology and management in the region.

Several limitations should be considered. First, only a limited number of studies were eligible for inclusion, restricting detailed subgroup analyses. Second, substantial heterogeneity between studies (I^2^ = 98.1%) limits the precision of the pooled incidence estimate despite the use of random-effects models and subgroup analyses. Third, publication bias could not be reliably assessed because of the small number of studies. Fourth, methodological quality varied across studies, and differences in study design, follow-up duration, and PKDL case definitions may have contributed to heterogeneity. Finally, most available evidence originated from Sudan, which may limit the generalizability of findings to other Eastern African countries. Additionally, country, treatment regimen, and operational era are strongly collinear in this literature, particularly for Sudanese studies conducted during the SSG-monotherapy period, preventing formal disentanglement of these effects.

## Conclusions

PKDL remains an important complication following VL treatment in Eastern Africa, with considerable variation in its occurrence across endemic settings. Treatment regimen emerged as the most important modifiable risk factor, while younger age, geographic location, and immune status were consistently associated with increased risk of disease development.

Recent evidence demonstrates excellent treatment outcomes with MF + PM combination therapy, supporting its consideration as a preferred treatment option in endemic settings. Strengthening post-treatment follow-up of VL patients, particularly among high-risk groups and in highly endemic areas, will be essential for early detection and management of PKDL. Future research should prioritize multicountry prospective studies using standardized case definitions and follow-up protocols to improve understanding of PKDL epidemiology and guide regional control strategies.

## Supporting information

S1 File PRISMA 2020 checklist for reporting the findings of the systematic review and meta-analysis on the incidence, risk factors, and treatment outcomes of post-kala-azar dermal leishmaniasis in East Africa. From: Page MJ, McKenzie JE, Bossuyt PM, Boutron I, Hoffmann TC, Mulrow CD, et al. The PRISMA 2020 statement: an updated guideline for reporting systematic reviews. BMJ 2021;372:n71. https://doi.org/10.1136/bmj.n71. This work is licensed under CC BY 4.0. To view a copy of this license, visit https://creativecommons.org/licenses/by/4.0/.
(PDF)

S2 File Detailed search strategy for the systematic review and meta-analysis on the incidence, risk factors, and treatment outcomes of post-kala-azar dermal leishmaniasis in East Africa. (PDF)

S3 File Data extraction table of included studies for the systematic review and meta-analysis on the incidence, risk factors, and treatment outcomes of post-kala-azar dermal leishmaniasis in East Africa. (PDF)

S4 File Risk of bias assessment of included studies, including Newcastle–Ottawa Scale (NOS) and Cochrane Risk of Bias 2 (RoB 2) tools, for the systematic review and meta-analysis on the incidence, risk factors, and treatment outcomes of post-kala-azar dermal leishmaniasis in East Africa. (PDF)

S1 Fig Leave-one-out sensitivity analysis for PKDL cumulative incidence. Each plot row displays the recalculated overall random-effects pooled estimate following the sequential omission of that specific cohort. Horizontal error bars represent the modified 95% confidence intervals.
(TIFF)

S2 Fig Contour-enhanced funnel plot of PKDL cumulative incidence. Standard errors are plotted against the logit-transformed proportions to evaluate study distribution symmetry. The vertical dashed line represents the central random-effects pooled estimate, while the shaded regions define the 95% and 99% pseudo-confidence contour boundaries used to differentiate reporting bias from clinical heterogeneity.
(TIFF)
