# Supplementary material for: From visceral to visible: A systematic review and meta-analysis of post-kala-azar dermal leishmaniasis incidence, risk factors, and treatment outcomes in Eastern Africa
Source: PLoS Negl Trop Dis. 2026 Aug 18;20(8):e0014666. doi: 10.1371/journal.pntd.0014666 (PMC13502647; doi:10.1371/journal.pntd.0014666)
Supplement: Supplementary file 2 [file pntd.0014666.s002.pdf]

From Visceral to Visible: A Systematic Review and Meta-Analysis of Post-Kala-Azar Dermal Leishmaniasis Incidence, Risk Factors, and Treatment Outcomes in Eastern Africa

Searching Path (n=1,927)

- PubMed (n=835)
- Cochrane Library (n=58)
- Scopus (n=783)
- AJOL (n=51)
- Google Scholar (n=200)*

****Note**: First 200 results screened as per standard grey literature practice for Google Scholar.*

PICOTS Table

This table maps each PICOTS element (Population, Intervention/Exposure, Comparator, Outcomes, Timing, Study design/Setting) to the corresponding eligibility criterion used in this review and to the specific search terms used to operationalize it across the five databases searched (PubMed, Cochrane Library, AJOL, Scopus, Google Scholar). Across all databases the search was built from three concept blocks joined with AND (OR used within each block, consistent with PRESS guidance): Block 1 = condition/disease terms, Block 2 = outcome and study-design indicator terms, Block 3 = geographic/setting terms. AJOL and Google Scholar searches omit or simplify Block 3 as noted in the search strategy document, since AJOL indexes only Africa-published journals and Google Scholar title-field searching already biases results toward the region.

PICOTS Element	Definition in This Review (per Eligibility Criteria)	Corresponding Search Terms	Search Block
Population	Patients with visceral leishmaniasis (VL) or post-kala-azar dermal leishmaniasis (PKDL), in East African countries (Ethiopia, Sudan, South Sudan, Kenya, Uganda, and neighboring endemic settings: Eritrea, Djibouti, Somalia, Tanzania).	Disease/condition terms: "Leishmaniasis, Visceral"[Mesh]; visceral leishmaniasis; kala-azar; kala azar; VL; "post-kala-azar dermal leishmaniasis"; PKDL; dermal leishmaniasis (combined with OR)<br>Geographic terms: "Africa, Eastern"[Mesh]; Ethiopia; Sudan; South Sudan; Kenya; Uganda; Eritrea; Somalia; Tanzania; "East Africa"; "Eastern Africa" (combined with OR)	Block 1 + Block 3
Intervention / Exposure	Primary VL treatment regimen received (e.g., SSG monotherapy, SSG+paromomycin, miltefosine+paromomycin); demographic and clinical risk factors (age, sex, HIV co-infection status, geographic sub-region).	risk factor; risk factors; predictor; determinant; hazard ratio; odds ratio; treatment; therap* (combined with OR; "Risk Factors"[Mesh] used in PubMed)	Block 2
Comparator	Alternate treatment regimen (e.g., combination therapy vs. SSG monotherapy) or absence of the	No dedicated comparator-specific search term; comparator status (where present) was extracted	— (screened, not searched)

	risk factor under study, where a comparator arm/group was reported. Not all eligible study designs have an explicit comparator (e.g., single-arm incidence cohorts), so this element was assessed at full-text screening rather than through a dedicated search term.	during data extraction, not filtered for at the search stage, to avoid excluding single-arm incidence/prevalence studies that are eligible for the incidence objective.	
Outcomes	(a) Cumulative incidence of PKDL among VL-treated patients; (b) risk-factor associations with PKDL occurrence; (c) PKDL treatment outcomes (cure, relapse, recurrence, treatment failure, response to therapy).	incidence; incidence rate; "Incidence"[Mesh]; "Treatment Outcome"[Mesh]; treatment outcome; cure; relapse; recurrence; failure; response; efficacy; effectiveness; outcome* (combined with OR)	Block 2
Timing	Any follow-up duration after VL treatment during which PKDL occurrence or treatment response was assessed; no minimum or maximum follow-up was imposed a priori. Actual follow-up duration was extracted per study and is reported in Table 1.	follow-up; longitudinal; prospective; retrospective; surveillance; registry (used as indicators of a defined observation period, not as an explicit date/duration filter)	Block 2
Study design / Setting	Cohort studies (prospective or retrospective) eligible for the incidence and treatment-outcome objectives; cohort and case-control studies eligible for the risk-factor objective; clinical trials, case series, and registries eligible where they report PKDL treatment response. Case-control and cross-sectional designs were not used to contribute to the pooled incidence estimate, as they cannot validly generate an incidence measure (see response to Reviewer #3, comment 4).	"Cohort Studies"[Mesh]; cohort; longitudinal; prospective; retrospective (used as design-indicator search terms; design eligibility for each specific objective was then confirmed during full-text screening, not by the search string alone)	Block 2

Note: exact search-string syntax differs slightly by database (e.g., [Mesh]/[tiab] in PubMed; ti,ab,kw in Cochrane Library; TITLE-ABS-KEY() in Scopus; intitle: in Google Scholar), but the underlying three-block PICOTS-aligned concept structure (Population/condition, Outcome/design, Setting/geography) shown above is applied consistently across all five databases. The full search strings for each database are provided below.

PubMed

("Leishmaniasis, Visceral"[Mesh] OR **"visceral leishmaniasis"[tiab]** OR **"kala-azar"[tiab]** OR **"kala azar"[tiab]** OR **VL[tiab]** OR "post-kala-azar dermal leishmaniasis"[tiab] OR "post kala azar dermal leishmaniasis"[tiab] OR PKDL[tiab] OR "dermal leishmaniasis"[tiab]) AND ("Incidence"[Mesh] OR "Risk Factors"[Mesh] OR "Epidemiology"[Mesh] OR "Cohort Studies"[Mesh] OR **"Treatment Outcome"[Mesh]** OR incidence[tiab] OR "incidence rate"[tiab] OR "risk factor"[tiab] OR "risk factors"[tiab] OR predictor*[tiab] OR "hazard ratio"[tiab] OR "odds ratio"[tiab] OR cohort[tiab] OR longitudinal[tiab] OR "follow-up"[tiab] OR prospective[tiab] OR retrospective[tiab] OR* ***"treatment outcome"[tiab]*** *OR relapse[tiab] OR* ***recurrence[tiab]*** *OR* ***efficacy[tiab]*** *OR* ***effectiveness[tiab]*** *OR* ***treatment[tiab]*** OR therap**[tiab]) AND ("Africa, Eastern"[Mesh] OR "Ethiopia"[Mesh] OR "Sudan"[Mesh] OR "Kenya"[Mesh] OR "Uganda"[Mesh] OR "South Sudan"[Mesh] OR Ethiopia[tiab] OR Sudan[tiab] OR Kenya[tiab] OR Uganda[tiab] OR Eritrea[tiab] OR Somalia[tiab] OR Tanzania[tiab] OR "South Sudan"[tiab] OR "East Africa"[tiab] OR "Eastern Africa"[tiab])

Cochrane Library

("post-kala-azar dermal leishmaniasis" OR "post kala azar dermal leishmaniasis" OR PKDL OR "post-kala-azar" OR "post-visceral leishmaniasis" OR "dermal leishmaniasis" OR "visceral leishmaniasis" OR "kala-azar" OR "kala azar"):ti,ab,kw

AND (incidence OR "incidence rate" OR "risk factor" OR "risk factors" OR predictor* OR determinant* OR "hazard ratio" OR "odds ratio" OR cohort OR longitudinal OR "follow-up" OR prospective OR retrospective OR surveillance OR registry **OR treatment OR therap* OR efficacy OR effectiveness OR cure OR relapse OR recurrence OR failure OR response OR outcome*):ti,ab,kw**

AND (Ethiopia* OR Sudan* OR Kenya* OR Uganda* OR Eritrea* OR Djibouti* OR Somalia* OR Tanzania* OR Rwanda* OR Burundi* OR "South Sudan" OR Malawi* OR Mozambique* OR Zambia* OR Zimbabwe* OR Madagascar* OR "East Africa" OR "Eastern Africa"):ti,ab,kw

AJOL

("post-kala-azar dermal leishmaniasis" OR "PKDL" OR "dermal leishmaniasis" OR "visceral leishmaniasis" OR "kala-azar")

AND (incidence OR "risk factors" OR cohort OR treatment OR efficacy OR cure OR outcomes)

(Note: Intentionally kept broad without a regional country filter block because AJOL exclusively indexes journals published natively within the African continent)

Scopus

TITLE-ABS-KEY("post-kala-azar dermal leishmaniasis" OR "post kala azar dermal leishmaniasis" OR "PKDL" OR "post-kala-azar" OR "post-visceral leishmaniasis" OR "dermal leishmaniasis" OR "visceral leishmaniasis" OR "kala-azar" OR "kala azar") AND TITLE-ABS-KEY("incidence" OR "incidence rate" OR "risk factor" OR "predictor" OR "determinant" OR "hazard ratio" OR "odds ratio" OR "cohort" OR "longitudinal" OR "follow-up" OR "prospective" OR "retrospective" OR "surveillance" OR "treatment" OR "therap*" OR "efficacy" OR "effectiveness" OR "cure" OR "relapse" OR "recurrence" OR "failure" OR "response" OR "outcome*") AND TITLE-ABS-KEY("Ethiopia" OR "Sudan" OR "Kenya" OR "Uganda" OR "Eritrea" OR "Djibouti" OR "Somalia" OR "Tanzania" OR "Rwanda" OR "Burundi" OR "South Sudan" OR "Malawi" OR "Mozambique" OR "Zambia" OR "Zimbabwe" OR "Madagascar*" OR "East Africa" OR "Eastern Africa")

Google scholar

(intitle:PKDL OR intitle:"post-kala-azar" OR intitle:"visceral leishmaniasis" OR intitle:"kala-azar") (incidence OR "risk factors" OR cohort OR treatment OR efficacy OR cure OR outcomes) (Ethiopia OR Sudan OR Kenya OR Uganda OR Eritrea OR Djibouti OR Somalia OR Tanzania OR Rwanda OR Burundi OR "South Sudan" OR "East Africa")<br>*Note: First 200 results screened as per standard grey literature practice for Google Scholar.*
