# Supplementary material for: From visceral to visible: A systematic review and meta-analysis of post-kala-azar dermal leishmaniasis incidence, risk factors, and treatment outcomes in Eastern Africa
Source: PLoS Negl Trop Dis. 2026 Aug 18;20(8):e0014666. doi: 10.1371/journal.pntd.0014666 (PMC13502647; doi:10.1371/journal.pntd.0014666)
Supplement: Supplementary file 3 [file pntd.0014666.s003.pdf]

DATA EXTRACTION TABLES

From Visceral to Visible: A Systematic Review and Meta-Analysis of Post-Kala-Azar Dermal Leishmaniasis Incidence, Risk Factors, and Treatment Outcomes in Eastern Africa

PROSPERO Registration: CRD420261411634

Table 1: Incidence Studies (n=5) | Table 2: Risk Factor Studies (n=5) | Table 3: Treatment Outcome Studies (n=5)

Table 1: Incidence Studies

Studies reporting PKDL incidence or rate after VL treatment in East African patients.

Author / Year	Country / Setting	Study Design	Study Period	Sample Size (n)	VL Treatment Received	PKDL Incidence (Primary Finding)	PubMed / DOI/Link
**Zijlstra EE** et al. 1994	Sudan Um-Salala village (endemic area)	Longitudinal prospective cohort (village-based)	April 1991 – April 1993 (2 years)	92 VL cases diagnosed; 85 followed ≥6 months	SSG standard regimen (dose varied per national guidelines)	**56% (48/85)** developed PKDL	https://pubmed.ncbi.nlm.nih.gov/7810819/
**Zijlstra EE** et al. 2000	Sudan Endemic village (kala-azar area)	Prospective village cohort (incidence arm) + cross-sectional clinical study	Not specified (follow-on from 1994 cohort area)	183 VL patients (incidence); 416 PKDL patients (clinical analysis)	SSG 20 mg/kg/day ×15 days (standard) OR inadequate/irregular treatment	**57% (105/183)** VL patients developed PKDL	https://pubmed.ncbi.nlm.nih.gov/10886148/
**Musa AM** et al. (AfriKADIA) 2022	Ethiopia, Sudan, Kenya, Uganda (4-country multicentre)	Phase III randomised controlled trial (non-inferiority)	2017 – 2021 (recruitment)	510 VL patients randomised (170 per arm; 3 arms)	Arm 1: MF+PM ×14 days Arm 2: SSG+PM ×17 days (Arm 3: extended PM+SSG)	**Overall: 28/340 (8.2%)** developed PKDL post-treatment<br>**MF+PM arm: 5/170 (2.9%)**<br>**SSG+PM arm: 23/170 (13.5%)**	DOI: 10.1093/cid/ciac643 Clin Infect Dis. 2023;76(3): e1177–e1185
**Tamiru A,** Mohammed R et al. 2021	Ethiopia University of Gondar LRTC, NW Ethiopia	Retrospective cohort (medical record review)	January 2012 – June 2019 (7 years)	2,836 VL patients treated; 1,000 included (SSG+PM); 280 with 6-month follow-up	SSG 20 mg/kg/day (max 850 mg/day) + PM 15 mg/kg/day ×17 days	**13/280 (4.6%)** developed PKDL at 6-month follow-up	https://pubmed.ncbi.nlm.nih.gov/34464401/
**Kennedy GC** et al. 2024	Kenya Tiaty East & West sub-counties	Retrospective cohort + cross-sectional follow-up	2018 – 2022 (hospital records + follow-up)	284 hospital records + 36 follow-up patients	SSG-based regimen (national Kenya guidelines)	**Follow-up cohort: 2/36 (5.56%)** PKDL<br>**Hospital records: 2/248 (0.80%)** PKDL	PMC: 11198830 PLOS ONE. 2024;19(6): e0306067

Table 2: Risk Factor Studies

Studies reporting risk factors independently associated with PKDL development in East African patients.

Author / Year	Country / Setting	Study Design	Study Period	Sample Size (n)	Risk Factors Examined	PubMed / DOI
**Musa AM** et al. (AfriKADIA) 2022	Ethiopia, Sudan (PKDL sub-analysis from 4-country RCT)	Phase III RCT sub-group analysis (pre-specified)	2017 – 2021	340 patients in PKDL-relevant arms (Ethiopia + Sudan sites)	VL treatment regimen (MF+PM vs SSG+PM), country (Sudan/Ethiopia vs Kenya/Uganda)	DOI: 10.1093/cid/ciac643 Clin Infect Dis. 2023;76(3): e1177
**Kimutai R,** Musa AM et al. 2017	Sudan (62.7%), Kenya (20.9%), Ethiopia (10.3%), Uganda (6.1%)	Prospective pharmacovigilance programme (SSG+PM first-line treatment)	2010 – 2014 (4-year programme)	3,126 VL patients enrolled (largest East Africa VL dataset)	HIV status, age (>50 yrs), country, primary vs relapse VL	https://pubmed.ncbi.nlm.nih.gov/28066878/
**Kennedy GC** et al. 2024	Kenya Tiaty sub-counties (rural endemic)	Retrospective cohort + cross-sectional (risk factor sub-analysis)	2018 – 2022	36 follow-up patients + 248 hospital records	Sex, geographic sub-county	PMC: 11198830 PLOS ONE. 2024;19(6): e0306067
**Zijlstra EE et al., 1994**	Sudan (Um-Salala village, Gedaref state, eastern Sudan)	Prospective longitudinal cohort study	April 1991 – April 1993	92 kala-azar cases diagnosed; 85 followed for ≥6 months for PKDL	Age (0-3 years higher risk for PKDL), sex, previous exposure to *Leishmania major* (protective effect against VL examined, not proven)	PMID: 7810819 / DOI: 10.4269/ajtmh.1994.51.826
**Zijlstra EE et al., 2000**	Sudan (Um-Salala village, Rahad province, Gedaref state, eastern Sudan)	Prospective cohort study	1991 – 1996	183 kala-azar patients (prospective study); 416 PKDL patients (clinical study)	Treatment adequacy (inadequate vs. standard SSG) [primary risk factor], age, sex, leishmanin skin test (LST) conversion	PMID: 10886148 / DOI: 10.1046/j.1365-2133.2000.03603.x

Table 3: Treatment Outcome Studies

Studies reporting efficacy and safety of anti-leishmanial regimens for confirmed PKDL in East African patients.

Author / Year	Country	Study Design	Study Period	Sample (n)	Regimen (Intervention vs Comparator)	Primary Outcome	PubMed / DOI
**Younis BM** et al.<br>2023	Sudan (Doka, El Hassan Centre)	Phase II open-label RCT (parallel arm, non-comparative)	2018–2021	110 randomised<br>104 completed<br>Age 6–60 yrs<br>Median age: 9 yrs<br>87% aged ≤12 yrs	**Arm 1 (PM/MF):**<br>**Arm 2 (LAmB/MF):**	**PM/MF: 54/55 (98.2%)** (95% CI 90.3–100)<br>**LAmB/MF: 44/55 (80.0%)** (95% CI 70.2–91.9)	PMCID: PMC10721181
**Abongomera C** et al. (MSF)<br>2016	South Sudan (Lankien, MSF programme)	Retrospective cohort (programme data 2002–2008)	2002 – 2008 (6 years)	422 severe PKDL<br>343: SSG mono<br>79: SSG+PM (all severe grade)	**SSG mono:**<br>**SSG+PM combo:**	**SSG mono: 90%**<br>**SSG+PM: 97%**	PMID: 27658288 PLOS ONE. 2016;11(9): e0163047
**Musa AM** et al.<br>2005	Sudan (Khartoum, institutional)	Prospective open-label pilot trial (single arm)	Not specified (pre-2005)	12 patients<br>Persistent PKDL ≥6 months SSG-refractory	**Intervention: Sodium Stibogluconate (SSG) combined with Leishvacin (an immunotherapy vaccine) versus SSG alone.**	**10/12 (83.3%)**	PMID: 16156969 Ann Trop Med Parasitol. 2005;99(6): 563–9
**Younis BM** et al.<br>2024	Sudan (Khartoum, University of Khartoum)	Phase IIb randomised double-blind placebo-controlled trial (NCT03969134)	2019 – 2022	86 randomised 40 vaccine 35 placebo 75 completed (87%)<br>Age 12–50 yrs<br>PKDL ≥6 months duration	**Intervention:**<br>**Comparator:**	**Vaccine: 6/40 (15%)**<br>**Placebo: 4/35 (11%)**<br>**p = 0.742 → NO EFFICACY**	PMID: 39253357 Mol Ther Methods Clin Dev. 2024;32(3): 101310
**El-Hassan AM** et al.<br>1992	Sudan (Institute of Endemic Diseases, Khartoum)	Retrospective clinical series (earliest treatment data)	Pre-1992	19 PKDL patients (clinical case series)	**SSG IV:**<br>**Ketoconazole:**	The response to intravenous sodium stibogluconate (20 mg/kg for 30 days) was reasonably good, although some patients required repeated or prolonged treatment. Ketoconazole at 10 mg/kg daily for 4 weeks had no effect on PKDL	PMID: 1329273 Trans R Soc Trop Med Hyg. 1992;86(3): 245–48
