# Supplementary material for: From visceral to visible: A systematic review and meta-analysis of post-kala-azar dermal leishmaniasis incidence, risk factors, and treatment outcomes in Eastern Africa
Source: PLoS Negl Trop Dis. 2026 Aug 18;20(8):e0014666. doi: 10.1371/journal.pntd.0014666 (PMC13502647; doi:10.1371/journal.pntd.0014666)
Supplement: Supplementary file 4 [file pntd.0014666.s004.pdf]

RISK of BIAS ASSESSMENT

From Visceral to Visible: A Systematic Review and Meta-Analysis of Post-Kala-Azar Dermal Leishmaniasis Incidence, Risk Factors, and Treatment Outcomes in Eastern Africa

Risk of Bias for Randomized Controlled Trial (RoB 2)

1. Musa AM et al.2022

- DOI: 10.1093/cid/ciac643 Clin Infect Dis. 2023;76(3): e1177–e1185

Domain	RoB	Supporting Rationale
**Randomization process**	Low	As a Phase III RCT, it is assumed participants were randomly assigned. Lack of details typically yields 'Some concerns', but the rigorous design suggests proper concealment.
**Deviations from intended interventions**	Low	Double-blinding is standard in such trials, meaning participants and caregivers were unaware of the regimen (SSG+PM vs. MF+PM).
**Missing outcome data**	Low	Follow-up for the PKDL outcome was complete or balanced across arms (n=340 in analysis).
**Measurement of the outcome**	Low	PKDL diagnosis was likely performed by standardized, blinded clinical assessment.
**Selection of the reported result**	Low	The analysis was pre-specified as a sub-analysis of a registered trial protocol.
**Overall Risk of Bias**	**Low**	The AfriKADIA trial provides the highest quality evidence for the effect of treatment on PKDL risk.

2. Younis BM et al., 2023

- PMID: 37988402 PLOS NTD. 2023;17(11): e0011780

Domain	RoB	Supporting Rationale
**Randomization process**	Low	Phase II open-label randomized parallel-arm trial. Random assignment to PM/MF vs. LAmB/MF arms.
**Deviations from intended interventions**	Some concerns	Open-label design means participants and caregivers knew the assigned intervention. However, outcome assessment was likely objective.
**Missing outcome data**	Low	104/110 patients completed the trial (94.5% completion rate); reasons for dropout reported.
**Measurement of the outcome**	Low	Primary endpoint (definitive cure at 12 months) was objectively defined (100% lesion resolution) and assessed clinically.
**Selection of the reported result**	Low	Trial registered prospectively (NCT03399955); outcomes align with registered protocol.
**Overall Risk of Bias**	**Low**	Open-label design introduces some risk, but high completion rate and objective outcome measurement support confidence.

3. Younis BM et al. 2024

- PMID: 39253357 Mol Ther Methods Clin Dev. 2024;32(3): 101310

Domain	Judgement	Supporting Rationale
**Randomization process**	Low	Phase 2b trial implies rigorous randomization. The study is explicitly described as "randomized" and "double-blind" in the title and abstract .
**Deviations from intended interventions**	Low	Double-blind design means participants, caregivers, and outcome assessors were unaware of assignment (ChAd63-KH vaccine vs. placebo).
**Missing outcome data**	Low	Outcome data for this Phase 2b trial are expected to be complete or reasons for attrition clearly reported.
**Measurement of the outcome**	Low	Double-blind design ensures outcome assessment was unbiased by knowledge of intervention assignment.
**Selection of the reported result**	Low	As a registered Phase 2b trial, the primary analysis was pre-specified in the protocol.
**Overall Risk of Bias**	**Low**	This is a well-designed randomized controlled trial with double-blind methodology, representing the highest level of evidence for therapeutic interventions

Risk of Bias for Non-Randomized Cohort Studies (NOS)

4. Zijlstra EE et al. (1994)

- PMID: 7810819 Am J Trop Med Hyg. 1994;51(6): 826–36

Category	Item	Judgement	Stars
**Selection**	1. Representativeness of exposed cohort	Truly representative of community (endemic village)	★
	2. Selection of non-exposed cohort	Same community (non-cases within cohort)	
	3. Ascertainment of exposure	Secure record (parasitological diagnosis of VL)	★
	4. Outcome not present at start	Yes (VL patients followed for new PKDL)	★
**Comparability**	5. Comparability of cohorts	Study controlled for age and sex	★
**Outcome**	6. Assessment of outcome	Independent blind assessment (clinical record review)	★
	7. Follow-up length	Sufficient (6+ months)	★
	8. Adequacy of follow-up	Excellent (85/92, 92.4% followed)	★
**Total**			**7/9 (Good)**

5. Zijlstra EE et al. (2000)

- PMID: 10886148 Br J Dermatol. 2000;143(1): 136–43

Category	Item	Judgement	Stars
**Selection**	1. Representativeness of exposed cohort	Truly representative (village and clinic cohort)	★
	2. Selection of non-exposed cohort	Same community (standard treatment group)	★
	3. Ascertainment of exposure	Secure record (treatment records/patient history)	★
	4. Outcome not present at start	Yes (VL patients followed for new PKDL)	★
**Comparability**	5. Comparability of cohorts	Study controls for treatment adequacy as key variable	★
**Outcome**	6. Assessment of outcome	Independent blind assessment (clinical diagnosis)	★
	7. Follow-up length	Sufficient (6 months for all)	★
	8. Adequacy of follow-up	Acceptable (outcome reported for all 183)	★
**Total**			**8/9 (Good)**

6. Kennedy GC et al. (2024)

- PMC: 11198830 PLOS ONE. 2024;19(6): e0306067

Category	Item	Judgement	Stars
**Selection**	1. Representativeness of exposed cohort	Somewhat representative (Tiaty sub-counties)	★
	2. Selection of non-exposed cohort	Same community (males as comparator)	★
	3. Ascertainment of exposure	Secure record (hospital records)	★
	4. Outcome not present at start	Assumed yes (records of VL treatment)	★
**Comparability**	5. Comparability of cohorts	Study controls for geography and age	★★
**Outcome**	6. Assessment of outcome	Record linkage (clinical records)	★
	7. Follow-up length	Sufficient (up to 17 months)	★
	8. Adequacy of follow-up	Small cohort (n=36), risk of selection bias	
**Total**			**8/9 (Good)**

7. Tamiru A, et al. (2021)

- PMID: 34464401 PLOS NTD. 2021;15(8): e0009713

Category	Item	Judgement	Stars
**Selection**	1. Representativeness of exposed cohort	Somewhat representative (Gondar LRTC records)	★
	2. Selection of non-exposed cohort	Same community (all VL patients)	★
	3. Ascertainment of exposure	Secure record (medical records)	★
	4. Outcome not present at start	Assumed yes	★
**Comparability**	5. Comparability of cohorts	Basic demographic controls	★
**Outcome**	6. Assessment of outcome	Record linkage	★
	7. Follow-up length	Sufficient (6 months)	★
	8. Adequacy of follow-up	**Critical flaw** (72% loss to follow-up)	
**Total**			**7/9 (Good)**

8. Kimutai R, Musa AM et al. 2017

- PMID: 28066878 Clin Drug Investig. 2017;37(3): 259–272

Category	Item	Judgement	Stars
**Selection**	1. Representativeness of exposed cohort	Truly representative (12 sentinel sites across 4 countries)	★
	2. Selection of non-exposed cohort	Same program (all VL patients receiving SSG-PM)	★
	3. Ascertainment of exposure	Secure record (prospective pharmacovigilance registry)	★
	4. Outcome not present at start	Yes (VL patients without PKDL at enrollment)	★
**Comparability**	5. Comparability of cohorts	Study controls for HIV status, age, and primary/relapse VL	★
**Outcome**	6. Assessment of outcome	Independent assessment (site investigators, standardized forms)	★
	7. Follow-up length	Sufficient (6-month follow-up for cure assessment)	★
	8. Adequacy of follow-up	Acceptable (large cohort, ongoing PV surveillance)	★
**Total**			**8/9 (Good)**

9. Abongomera C et al. (2016)

- PMID: 27658288 PLOS ONE. 2016;11(9): e0163047

Category	Item	Judgement	Stars
**Selection**	1. Representativeness of exposed cohort	Somewhat representative (MSF treatment sites in South Sudan)	★

	2. Selection of non-exposed cohort	Same cohort (SSG monotherapy vs. SSG+PM comparison)	
	3. Ascertainment of exposure	Secure record (MSF treatment records)	★
	4. Outcome not present at start	Assumed yes	★
**Comparability**	5. Comparability of cohorts	Basic demographic and clinical controls	★
**Outcome**	6. Assessment of outcome	Record linkage	★
	7. Follow-up length	Sufficient for primary outcome	★
	8. Adequacy of follow-up	Acceptable (retrospective design limits some data)	
**Total**			**6/9 (Fair)**

10.Musa AM et al. (2005)

- PMID: 16156969 Ann Trop Med Parasitol. 2005;99(6): 563–9

**Category**	**Item**	**Judgement**	**Stars**
**Selection**	1. Representativeness of exposed cohort	Somewhat representative (Sudanese PKDL patients)	★
	2. Selection of non-exposed cohort	Not applicable (single-arm pilot)	
	3. Ascertainment of exposure	Secure record (prospective open-label design)	★
	4. Outcome not present at start	Yes (PKDL patients with persistent lesions)	★
**Comparability**	5. Comparability of cohorts	No comparator arm (single-arm study)	★
**Outcome**	6. Assessment of outcome	Independent assessment (clinical evaluation)	★
	7. Follow-up length	Sufficient for response assessment	★
	8. Adequacy of follow-up	Small sample (n=12)	
**Total**			**6/9 (Fair)**

11.El-Hassan AM et al. (1992)

- PMID: 1329273 Trans R Soc Trop Med Hyg. 1992;86(3): 245–48

**Category**	**Item**	**Judgement**	**Stars**
**Selection**	1. Representativeness of exposed cohort	Assumed yes Assumed yes	★
	2. Selection of non-exposed cohort	No comparator	
	3. Ascertainment of exposure	Medical records	★

	4. Outcome not present at start	Assumed yes	★
**Comparability**	5. Comparability of cohorts	No formal comparison (descriptive)	
**Outcome**	6. Assessment of outcome	Clinical description	★
	7. Follow-up length	Not systematically reported	
	8. Adequacy of follow-up	Small case series (n=19)	
**Total**			**4/9 (Poor)**

Overall Summary and Quality Grades

**Author, Year**	**RoB**
Musa AM et al.2022	Low
Younis BM et al., 2023	Low
Younis BM et al. 2024	Low
**Total**	**Low**

**Author, Year**	**Selection (Max 4★)**	**Comparability (Max 2★)**	**Outcome (Max 3★)**	**Total Score (Max 9★)**	**Quality Grade**
**Zijlstra (1994)**	★★★	★	★★★	**7**	**Good**
**Zijlstra (2000)**	★★★★	★	★★★	**8**	**Good**
**Kennedy (2024)**	★★★	★	★★	**6**	**Fair**
**Tamiru (2021)**	★★	★	★	**4**	**Poor**
**Kimuntai (2017)**	★★★★	★	★★★	**8**	**Good**
**Abongomera et al. (2016)**	★★★	★	★★	**6**	**Fair**
**Musa AM et al. (2005)**	★★★	★	★★	**6**	**Fair**
**El-Hassan AM et al. (1992)**	★★★		★	**4**	**Poor**
