# Supplementary material for: From visceral to visible: A systematic review and meta-analysis of post-kala-azar dermal leishmaniasis incidence, risk factors, and treatment outcomes in Eastern Africa
Source: PLoS Negl Trop Dis. 2026 Aug 18;20(8):e0014666. doi: 10.1371/journal.pntd.0014666 (PMC13502647; doi:10.1371/journal.pntd.0014666)
Supplement: Supplementary file 5 [file pntd.0014666.s008.docx]

# Point-by-point response

Dear Editor-in-Chief,

We thank both reviewers for their careful, constructive, and expert reading of the manuscript. The comments have identified important places where the link between our findings and the review objectives needs to be made explicit, where results pertaining to primary VL treatment and PKDL treatment were insufficiently distinguished, and where regional epidemiological context (parasite strain, vector, host genetics, HIV prevalence, and historical treatment quality) needed fuller discussion. We have carefully addressed all feedback and have prepared a point-by-point response, with revisions highlighted in green within the manuscript. The manuscript has been updated accordingly, to reflect these changes.

# Journal Requirements

1) Please ensure that the CRediT author contributions listed for every co-author are completed accurately and in full. At this stage, the following Authors/Authors require contributions: Eyob Girma Abera. Please ensure that the full contributions of each author are acknowledged in the "Add/Edit/Remove Authors" section of our submission form. The list of CRediT author contributions may be found here:

<https://journals.plos.org/plosntds/s/authorship#loc-author-contributions>

***Response****: Thank you. Yes, we have confirmed, all are correct.*

2) Please provide an Author Summary. This should appear in your manuscript between the Abstract (if applicable) and the Introduction, and should be 150-200 words long. The aim should be to make your findings accessible to a wide audience that includes both scientists and non-scientists. Sample summaries can be found on our website under Submission Guidelines:

<https://journals.plos.org/plosntds/s/submission-guidelines#loc-parts-of-a-submission>

***Response****: Thank you, we have added the section in our manuscript accordingly.*

3) We do not publish any copyright or trademark symbols that usually accompany proprietary names, eg ©, ®, or TM (e.g. next to drug or reagent names). Therefore please remove all instances of trademark/copyright symbols throughout the text, including: - ® on page: 32.

***Response****: Thank you. We have removed the marks from the document. If we have missed any, please let us know. Alternatively, we acknowledge and approve their removal by your side on our behalf.*

4) We have amended your Competing Interest statement to comply with journal style. We kindly ask that you double check the statement and let us know if anything is incorrect.

***Response****: Thank you, we accept the amendment and approved.*

# Reviewer #1:

## General comment

1. The authors conducted a systematic literature review on PKDL. In particular they looked at risk of PKDL after VL treatment, taking into account possible associated factors such as treatment regimen for VL, geographic site, age, sex. They conclude that there’s a higher risk for PKDL in East Africa, though heterogeneity within the region is substantial. Highest risk was found in Sudan, young age was also a risk factor. Treatment of primary VL episode was a key risk factor, with PKDL risk decreasing as SSG monotherapy was abandoned. They also looked at treatment of CL, MF+PM seemed very promising.

***Response****: Thank you for the valuable feedback. We have revised the manuscript to further clarify these findings and emphasize the key factors associated with PKDL risk, including geographic variation, age, and VL treatment regimens.*

2. This is a well written and interesting manuscript, there are just some things that could be better clarified. In particular it is not always clear how findings are linked to the main objectives. For results on treatment it’s not always clear whether it’s about treatment of PKDL or of the original VL episode. Also at times it appears risk factors overlap, i.e. Sudan and SSG monotherapy. It would be good to disentangle these effects or explain why this was not possible.

***Response****: Thank you for the feedback. We agree that every treatment-related finding should clearly specify whether it refers to the primary VL episode or PKDL treatment. In the revised manuscript, we have added clarifying subheadings and opening sentences in both the Results and Discussion sections to distinguish these. Regarding Sudan and SSG overlap, this remains a major confounding factor in the available literature. Most pre-2010 Sudanese cohorts used SSG monotherapy, while post-2010 multi-country studies mainly reflect combination regimens, resulting in strong overlap between country, study period, and treatment regimen. Since no included study provided individual-level data to separate these effects statistically, we have clarified this limitation in the Discussion and Limitations sections and interpreted the findings within the available evidence.*

3. Line 257-259: ‘Sudan emerged as the clear high-risk epicenter with an exceptional pooled PKDL cumulative incidence of 56.7%’. Comment: Does this difference still persist after controlling for treatment regimen?

***Response****: Thank you for comment. We cannot formally control for regimen at the patient level because no included Sudanese study reported regimen-stratified incidence permitting adjustment. However, almost all Sudanese studies in this review were conducted during the SSG-monotherapy era, meaning country and treatment regimen are near-perfectly collinear at the study level. More recent supervised, quality-controlled treatment data from Sudan with SSG+PM combination therapy reported substantially lower PKDL incidence (1-2%) under routine conditions [Atia et al. 2015], supporting that treatment adequacy, not merely country, is the operative factor. We have clarified this interpretation in the Discussion section.*

4. Line 346-349: ‘On a localized scale, recent field data from rural Kenya highlighted intense micro-geographical clustering, demonstrating that risk boundaries vary significantly between adjacent administrative sectors, such as Tiaty East versus Tiaty West (p=0.03) [19]’. Comment: is this about risk for PKDL or risk for VL? If it is about PKDL, did you adjust for VL incidence?

***Response****: Thank you for this important clarification. Upon re-checking the source study (Kennedy et al. 2024), the finding reflects combined VL follow-up and PKDL outcomes, and the p=0.03 comparison was not adjusted for underlying VL incidence by sub-county. We have revised the text to clarify that this comparison was not adjusted for VL transmission intensity and therefore cannot be interpreted as a PKDL-specific spatial effect independent of VL risk.*

5. Line 357-358: ‘co-infected patients had an initial cure rate of only 56% compared to 95.1% overall’. Comment: Again not clear if this is about VL or PKDL, if it is about VL, does it belong in this manuscript?

***Response****: Thank you for this clarification. This finding refers to cure of the primary VL episode, not PKDL treatment. We retain it because incomplete VL clearance in HIV-co-infected patients is a plausible upstream pathway to PKDL. We have revised the sentence to explicitly state that the 56% and 95.1% figures refer to initial VL cure rates, making it clear this is a risk factor context rather than a PKDL treatment result.*

6. Line 446-447: ‘The between-study variance was demonstrably attributable to country-level differences, study design, and operational era rather than random sampling error.’ Comment: Was it not the treatment regimen used in the VL episode that was the most important factor (as mentioned in lines 463-469)?

***Response****: Thank you for this important observation. We agree this reads as contradictory. Country, study design, and operational era are not independent of treatment regimen in this literature; they are proxies for it, since regimen use is strongly patterned by country and calendar period. We have revised the text to clarify that these factors are interrelated and best understood as markers of which regimen was in use rather than independent causal drivers.*

7. Line 491-493: ‘Sudanese longitudinal cohorts documented that infants and toddlers aged 0–3 years faced a cumulative PKDL risk of 78%–81% under historical antimonial environments [16,17].’ Comment: Is there also evidence that older age groups in this cohort that were treated with the same regimen had significantly lower risk of PKDL?

***Response****: Yes. In the same Sudanese longitudinal cohort, PKDL was markedly more frequent in the very young: 7 of 9 (78%) VL cases aged 0–1 year and 13 of 16 (81%) aged 2–3 years developed PKDL, whereas among all VL cases in the cohort (mean age 6 years) the overall PKDL proportion was 56%. This indicates that older children and adults treated with the same antimonial regimen in the same setting had a substantially lower cumulative risk of PKDL, consistent with a strong within‑cohort age gradient rather than a between‑era effect. We have updated the manuscript accordingly.*

8. Line 507-509: ‘our review uncovered compelling evidence of micro geographical PKDL clustering, with significant incidence variation between adjacent administrative sectors in Kenya (Tiaty East vs. Tiaty West; p=0.03) [19]’. Comment: See earlier comment, did you take into account the clustering of primary VL? If you explain it by vector ecology (line 510-511) then it is about VL clustering in the first place.

***Response****: Thank you for this important comment. We agree with your observation. If the mechanism is vector ecology, the effect most plausibly operates on VL transmission rather than PKDL risk conditional on VL. The source study did not adjust for underlying VL incidence, so this finding cannot be interpreted as PKDL-specific. We have revised the Discussion to soften the causal claim and reframe the recommendation as combined VL/PKDL surveillance rather than PKDL-specific clustering.*

## Minor comment

9. Line 40: ‘heterogeneity was assessed using the I² statistic’. Comment: Please provide a reference.

***Response****: Thank you for this suggestion. We have now added the appropriate reference for the I² statistic.*

10. Line 158: ‘Data on incidence were extracted and pooled using random-effects meta-analysis’. Comment: Please specify which level of random effects, study, country, both?

***Response****: Thank you for this clarification. Random effects were modelled at the individual-study level (study-specific proportions pooled with the REML estimator for τ²). Country was used only as a subgroup moderator in descriptive subgroup analyses, not as a nested random-effects level, since too few studies existed per country to support that.We have updated the method section to clarify that.*

11. Line 248: ‘Among the total 924 patients, the pooled cumulative incidence of PKDL among treated visceral leishmaniasis patients in East Africa was 18.2% (95% CI: 4.7%–49.7%)’ Comment: Not clear where the 924 comes from since in the paragraph above it is stated that the total sample size was 4,699.

***Response****: 4,699 is the total across all 11 included studies (incidence + risk-factor + treatment studies); 924 is the subset from only the five studies contributing cumulative-incidence data. This is detailed under the 'Characteristics of Included Studies' section along with the Table 1 and also S3 File. We have revised the sentence to clarify this.*

12. Line 325-328: ‘Conversely, data from the 3,126-patient prospective pharmacovigilance registry indicated that advanced age over 50 years acts as a protective factor, demonstrating significantly higher cure rates (81.4%) compared to younger patients (95.1% overall) [9]’. Comment: What is meant here by cure rate and why is the cura rate higher for younger patients if older age is a protective factor? Why is this mentioned in the section of risk factors for PKDL?

***Response****: Thank you for this correction. We have revised the text to accurately reflect what Kimutai et al., 2017 reports: older patients (>50 years) had a lower initial VL cure rate (81.4%) compared to the overall cure rate (95.1%), meaning older age is a risk factor for poorer VL treatment outcomes, not a protective factor. We have corrected this error.*

# Reviewer #2:

The manuscript provides a systematic review and meta-analysis on aspects of PKDL using PRISMA 2020 guidelines and JBI methodology.

## General comments

While the methodology seems sound, the results do not represent the published literature and therefore deviate from what we know on the epidemiology and pathophysiology of VL and PKDL in the region, as this was not captured in the papers examined.

***Response****: Thank you. This regional context is well established but under-represented in our Discussion because it wasn't quantitatively reported in the 11 included studies. We add a new Discussion subsection drawing on the wider literature to explain why our pooled/subgroup estimates need cautious interpretation, addressing each specific point (1–8) below.*

1. Differences in VK in East Africa should be clear
a. From the older literature, the parasite is L. donovani but the strain in Sudan / Northern Ethiopia is different from that found in South Ethiopia / Kenya.
b. Also from the older literature, the vector is different: in Sudan /Northern Ethiopia P. orientalis, while in South Ethiopia / Kenya it is mainly P. martini.
c. However, in the recent outbreak in Kenya the L. donovani that is circulating may be yet another subtype and P. orientalis may play a more important role with higher infection rates. It is however not clear if these findings are really new or may not have been detected in previous years.

***Response****: Thank you for this important contextual information. We agree that the region is not epidemiologically homogeneous, and strain and vector differences are plausible biological drivers of the geographic heterogeneity our pooled analysis could only describe statistically. We have added a paragraph in the new Discussion subsection acknowledging that different L. donovani strains and different dominant sandfly vectors exist between Sudan/Northern Ethiopia (P. orientalis) and Southern Ethiopia/Kenya (P. martini), and that whether the parasite subtype and vector pattern recently reported in the Kenyan outbreak represent a genuinely new combination or a previously undetected one remains unresolved.*

2. The genetic background of the population is important: even in the endemic area in Sudan there are differences in PKDL rates between villages that basically share the same ecology with the Masaleet as the most affected tribe. Clearly, as a whole the Sudanese and South Sudanese populations are different from those in Ethiopia and Kenya.

***Response****: Thank you for highlighting this important aspect. We agree that host genetic susceptibility is a recognized contributor to residual heterogeneity that our review could not quantify, since no included study reported host genotyping or ethnicity-stratified incidence. We have added a paragraph in the new Discussion subsection acknowledging that host genetic susceptibility to PKDL varies both within and between countries, with reported differences in PKDL rates between Sudanese villages sharing similar ecology and certain ethnic groups disproportionately affected, and that none of the included studies reported host genotyping or ethnicity-stratified incidence.*

3. On the same note HIV co-infection in VL is common in Ethiopia while in Sudan it is rarely found.

***Response****: Thank you for this important observation. We agree that HIV co-infection prevalence among VL patients differs substantially across Eastern African countries, being common in Ethiopia but rarely reported in Sudanese cohorts. Because our HIV-related findings are drawn predominantly from the multi-country pharmacovigilance registry, they should not be assumed to generalize uniformly across countries with very different underlying HIV-VL overlap. We have added a paragraph in the new Discussion subsection acknowledging this limitation.*

4. The studies examined differ in design and follow-up time. For example the RCTs quoted have strict entry criteria while the clinical studies are likely to reflect the true situation. In addition, in the RCTs the follow-up time is 6 months while it is clear that PKDL in Sudan mostly occurs between 0-13 months after VL treatment.

***Response:*** *Thank you for this important methodological observation. We agree that study design and follow-up duration significantly influence PKDL incidence estimates. RCTs with strict entry criteria and 6-month follow-up, such as the AfriKADIA trial, will systematically under-ascertain PKDL cases compared to clinical and cohort studies with longer follow-up. Since PKDL in Sudan occurs predominantly 0-13 months after VL treatment, incidence estimates from trials with limited follow-up should be interpreted as minimum estimates, while the higher estimates from longer-following cohort studies are likely closer to true population incidence. We have added a paragraph in the new Discussion subsection acknowledging this limitation and have also included it as a formal item in the Limitations section.*

5. The high PKDL rate that was reported from Sudan in early studies was caused by adulterated (mixed with water) SSG administered by unqualified health workers in too low dosage and intermittent duration. This suggested undertreatment as an important risk factor for PKDL. Subsequent studies with supervised treatment and proper follow-up showed much lower PKDL rates and the last available study showed 20-28%.

***Response****: Thank you for this important clarification. We agree that the very high early Sudanese PKDL rates were largely driven by sub-standard, unsupervised SSG administration rather than SSG as a drug choice. We have added a paragraph in the new Discussion subsection acknowledging that subsequent supervised, quality-controlled treatment data from Sudan reported substantially lower incidence, supporting treatment adequacy as the key modifiable driver.*

6. The resistance issue towards SSG should be discussed on how it differs between Asia and Africa, also with regard to PKDL incidence.

***Response****: Thank you for this suggestion. We have added a brief contrast in the new Discussion subsection noting that unlike the Indian subcontinent, where widespread clinical SSG resistance drove the shift away from antimonial monotherapy, East African L. donovani has not shown comparable levels of documented SSG resistance, and the regional decline in SSG use appears driven predominantly by toxicity, adherence, and treatment-supervision concerns.*

7. What is the explanation for the differences between males and females with regard to PKDL risk? Mentioning this is only relevant if you present potential explanations.

***Response****: Thank you for this important observation. We agree that reporting an association without mechanism is not useful. None of the included studies tested explanatory mechanisms directly. We have revised the Discussion to state that the direction of this association is inconsistent across settings (male predominance in Sudan and Ethiopia; female association in Kenyan data) and that commonly proposed but unverified explanations include differential outdoor or occupational sandfly exposure and differential health-care-seeking behaviour by sex. We flag these as hypotheses for future studies rather than established findings.*

8. The issue of persisting parasites in the skin after VL treatment is not discussed as well as PK/PD issues in VL treatment.

***Response****: Thank you for this important mechanistic point. We agree that this gap should be explicitly named. We have added a paragraph in the new Discussion subsection noting that PKDL is increasingly understood to reflect persistence of viable Leishmania parasites in the skin despite systemic clearance of VL, potentially reflecting sub-therapeutic drug penetration into dermal tissue, and that pharmacokinetic and pharmacodynamic studies of drug penetration into skin represent a priority for future research.*

## Specific comments

1. Line 61: not only L. donovani

***Response****: Thank you for this correction. We agree that the opening sentence over-simplified by attributing all regional VL to one undifferentiated parasite. We have revised the sentence to specify that VL in Eastern Africa is caused predominantly by Leishmania donovani, with regional strain variation across the region.*

2. Line 76: ref 7 may not be the correct one

***Response:*** *Thank you for confirming. Upon verification, the reference you cited (Zijlstra et al., 2017) is indeed the correct source for the statement about South Asian PKDL affecting adolescents and adults years after VL cure. No revision to the manuscript is needed.*

3. Line 131-4: this is probably the problem in the methodology

***Response****: Thank you for this important observation. We agree that the aggregation of age, country, era, and regimen at the study level rather than patient level is a core limitation preventing multivariable adjustment. We have added an explicit statement in the Methods section acknowledging this limitation. Since S3 File already contains the relevant study-level data (country, regimen, and era), no additional supplementary table is needed to illustrate this point.*

4. Line 304: disease velocity?

***Response****: Thank you for this comment. We agree that "disease velocity" is a non-standard phrase. We have replaced it with clearer terminology.*

5. Line 337-351: this needs much better analysis and discussion

***Response****: Thank you for this comment. We have addressed the Tiaty analysis and discussion through the revisions made for the earlier comments on this finding. In the Results section, we have clarified that this reflects combined VL/PKDL follow-up outcomes, not PKDL-specific clustering. In the Discussion section, we have added the caveat that the source study did not adjust for underlying VL incidence and reframed the implication as supporting combined VL/PKDL surveillance rather than a PKDL-specific spatial effect. No additional action is needed.*

6. Line 444: this needs to be discussed in detail. The ref seems not correct.

***Response:*** *Thank you for this comment. We have expanded the discussion of the geographic variation between Ethiopia, Sudan, and multi-country registries by elaborating on the heterogeneous ecological, parasite-strain, host-genetic, and treatment-related drivers across the region in the new Discussion paragraph. Regarding the reference concern, we have verified and corrected the citation. The reference [17] has been updated to the correct source: Zijlstra EE. Risk factors for post-kala-azar dermal leishmaniasis (PKDL): Challenges in understanding pathophysiology. PLoS Negl Trop Dis. 2026;20(2):e0013952.*

7. Line 462 and 469: there should be better refs for this than Proceedings of this meeting

***Response****: Thank you for the observation. We agree that the original citation did not directly support the specific country-level and multi-country estimates. We have therefore revised the sentence to avoid unsupported numerical claims and to cite literature that explicitly documents regional heterogeneity in PKDL incidence across Eastern Africa and the Indian subcontinent. We have also replaced the meeting report in the discussion on treatment-related changes with a peer-reviewed study from Bihar showing that increased amphotericin B use coincided with a decline in PKDL cases.*

8. Line 492: what are historical antimonial environments? Please avoid phrases or concepts that are not common language.

***Response****: Thank you for this comment. We agree that "historical antimonial environments" is not a standard phrase. We have revised the text to use clearer language.*

# Reviewer #3

## Major

1. Incoherence between search strategy and study eligibility criteria

Line 124-125 of the eligibility criteria says: “Studies were included if they involved patients with VL or PKDL in East African countries, including Ethiopia, Sudan, Kenya, Uganda, and neighboring endemic settings”

The search term presented on supplement file2 uses AND instead. Instead of finding any studies with VL or PKDL, the use of AND will only restrict the search to the VL studies also reporting PKDL. For example, If I use the following search on pubmed, it returned 826 hits (15th July 2026). And this is not as comprehensive list of countries as used by the study authors.

( "Leishmaniasis, Visceral"[Mesh] OR "Post-Kala-Azar Dermal Leishmaniasis"[Mesh] OR "visceral leishmaniasis"[tiab] OR "visceral leishmaniases"[tiab] OR "kala azar"[tiab] OR kala-azar[tiab] OR VL[tiab] OR "post-kala-azar dermal leishmaniasis"[tiab] OR "post kala azar dermal leishmaniasis"[tiab] OR PKDL[tiab] ) AND ( Sudan[Mesh] OR "South Sudan"[Mesh] OR Ethiopia[Mesh] OR Kenya[Mesh] OR Uganda[Mesh] OR Sudan[tiab] OR "South Sudan"[tiab] OR Ethiopia[tiab] OR Kenya[tiab] OR Uganda[tiab] ) AND ( incidence[tiab] OR inciden*[tiab] OR epidemiolog*[tiab] OR prevalence[tiab] OR frequency[tiab] OR burden[tiab] OR "risk factor*"[tiab] OR predictor*[tiab] OR determinant*[tiab] OR associat*[tiab] OR prognos*[tiab] OR "treatment outcome*"[tiab] OR outcome*[tiab] OR treatment[tiab] OR therap*[tiab] OR efficacy[tiab] OR effectiveness[tiab] OR response[tiab] OR cure[tiab] OR relapse[tiab] OR recurrence[tiab] OR failure[tiab] OR follow-up[tiab] )

I am not quite convinced (or it is not clear to me from reviewing the manuscript) that the study authors have designed the search term to align with eligibility criteria. It would be good to clarify the PICOTS elements of the search. I would like the authors to clarify this.

***Response:*** *We thank the reviewer for this important observation, which prompted a substantial revision of our search strategy. We revised Block 1 to add explicit free-text terms ("visceral leishmaniasis," "kala-azar," "VL") alongside the existing MeSH terms, and expanded Block 2 to include the full range of treatment-outcome terms (treatment, therapy, efficacy, effectiveness, cure, relapse, recurrence, failure, response) in addition to the incidence and risk-factor terms already present, while retaining the PRESS-consistent block logic (OR within concept blocks, AND across blocks) and adding an explicit PICOTS table to make this structure transparent. Re-running the expanded search across all databases increased the total number of records identified from 532 to 1,927.*

*We then re-screened all newly identified records against our pre-specified eligibility criteria. Although the yield of records nearly quadrupled, no additional studies met the eligibility criteria beyond the 11 originally included, and the PRISMA flow diagram has been updated accordingly to reflect the larger initial pool alongside the unchanged final study set. We view this as a reassuring result rather than a null one; it indicates that our original search, despite its narrower construction, was not systematically missing eligible studies. The published literature on PKDL incidence, risk factors, and treatment outcomes in Eastern Africa is genuinely limited to the 11 studies analyzed, which strengthens rather than undermines confidence in the completeness of the evidence synthesized in this review.*

## Other comments

2. Abstract Results.

“The pooled cumulative PKDL incidence was 18.2% (95% CI: 4.7%–49.7%), with substantial heterogeneity (I² = 98.1%)”

The time of PKDL appearance is important, but this is currently lacking on the manuscript. Please present the time-frame. Are these within 3-months, 6-months, or 1year of treatment?

***Response****: We thank the reviewer for this important observation. We agree that the time frame of PKDL appearance is clinically and epidemiologically important. The included studies varied in their follow-up durations: the Zijlstra 1994 and 2000 Sudanese cohorts followed patients for up to 12 months or longer, reflecting the 0-13 month window during which PKDL typically appears in Eastern Africa. In contrast, the AfriKADIA RCT and other more recent trials had fixed 6-month follow-up periods, while the Kennedy 2024 Kenyan study had variable follow-up ranging from 10 to 24 months (mean 17 months) among active follow-up cases. These differences likely influence reported incidence estimates, with shorter follow-up periods potentially underestimating true incidence. We have revised the Abstract and main Results sections to make this transparent.*

3. “Significant geographic variation was observed, with Sudan reporting the highest pooled incidence (56.7%) compared to Ethiopia (4.6%) and multi country cohorts (8.2%)”

But when? Recent studies from Sudan indicate not a very high PKDL incidence. Temporal elements remain important in the context of infectious diseases, due to periodic changes in first line drugs used over time.

***Response****: We thank the reviewer for this important observation. We agree that temporal elements are critical, as first-line VL treatment regimens have changed over time, directly influencing PKDL incidence. The 56.7% figure for Sudan reflects the historical SSG-monotherapy era (1994-2000), while more recent supervised treatment data from Sudan and multi-country cohorts (2021-2024) show substantially lower incidence. We have revised the Abstract to include the specific study periods alongside the geographic finding.*

4. Main text, Methodology

The review included observational studies (cohort, case-control, and cross-sectional studies). The authors stated that studies reporting PKDL incidence, risk factors, or treatment outcomes among VL patients in Eastern Africa were eligible. Not all the observational studies are suitable for answering questions regarding incidence, risk factors or outcomes. For example, case-control remain typically unsuitable to assess disease incidence.

I suggest the authors clearly presenting what designs were considered for identifying studies related to each of the research objectives.

***Response****: We thank the reviewer for this important methodological observation. We agree that not all observational study designs are equally suitable for answering each research question. Case-control studies, for example, are inappropriate for assessing disease incidence. To address this, we have revised the Methods section to clearly specify which study designs were considered for each research objective: cohort studies (both prospective and retrospective) for incidence estimation; cohort and case-control studies for risk factor identification; and cohort studies and clinical trials for treatment outcome assessment. This clarification ensures transparency in how evidence was matched to each objective, while we also described this in detail in the data extraction supplementary file.*

5. Table 1

a) Clearly indicate the follow-up duration of the studies

b) Clearly indicate if these studies are among VL patients or among PKDL patients?

c) There is little info on study meta-data. Not clear what “weights” (%) are referring to here. Weights for the study for the meta-analysis?

***Response****: We thank the reviewer for these valuable suggestions. For readability, we have kept Table 1 in its current presentation format, but we have added a narrative description of the follow-up duration for the five incidence studies in the Results section. We have also directed readers to S3 File for complete study-level details. Regarding the weights, we have moved their description to the Figure 2 legend, where they are visually represented.*
