# Supplementary material for: From visceral to visible: A systematic review and meta-analysis of post-kala-azar dermal leishmaniasis incidence, risk factors, and treatment outcomes in Eastern Africa
Source: PLoS Negl Trop Dis. 2026 Aug 18;20(8):e0014666. doi: 10.1371/journal.pntd.0014666 (PMC13502647; doi:10.1371/journal.pntd.0014666)
Supplement: Peer review history

# Peer review: Decision Letter 0

**DOI:** 10.1371/journal.pntd.0014666.r001

**Authors:** Hira Nakhasi, Mitali Chatterjee

21 Jul 2026

PNTD-D-26-01113

From Visceral to Visible: A Systematic Review and Meta-Analysis of Post-Kala-Azar Dermal Leishmaniasis Incidence, Risk Factors, and Treatment Outcomes in Eastern Africa

PLOS Neglected Tropical Diseases

Dear Dr. Abera,

Thank you for submitting your manuscript to PLOS Neglected Tropical Diseases. After careful consideration, we feel that it has merit but does not fully meet PLOS Neglected Tropical Diseases's publication criteria as it currently stands. Therefore, we invite you to submit a revised version of the manuscript that addresses the points raised during the review process.

Please submit your revised manuscript within by Sep 19 2026 11:59PM. If you will need more time than this to complete your revisions, please reply to this message or contact the journal office at plosntds@plos.org. When you're ready to submit your revision, log on to https://www.editorialmanager.com/pntd/ and select the 'Submissions Needing Revision' folder to locate your manuscript file.

Please include the following items when submitting your revised manuscript:

* A letter that responds to each point raised by the editor and reviewer(s). You should upload this letter as a separate file labeled 'Response to Reviewers'. This file does not need to include responses to any formatting updates and technical items listed in the 'Journal Requirements' section below.

* A marked-up copy of your manuscript that highlights changes made to the original version. You should upload this as a separate file labeled 'Revised Manuscript with Track Changes'.

* An unmarked version of your revised paper without tracked changes. You should upload this as a separate file labeled 'Manuscript'.

If you would like to make changes to your financial disclosure, competing interests statement, or data availability statement, please make these updates within the submission form at the time of resubmission. Guidelines for resubmitting your figure files are available below the reviewer comments at the end of this letter.

As the corresponding author, your ORCID iD is verified in the submission system and will appear in the published article. PLOS supports the use of ORCID, and we encourage all coauthors to register for an ORCID iD and use it as well. Please encourage your coauthors to verify their ORCID iD within the submission system before final acceptance, as unverified ORCID iDs will not appear in the published article. Only the individual author can complete the verification step; PLOS staff cannot verify ORCID iDs on behalf of authors.

We look forward to receiving your revised manuscript.

Kind regards,

Mitali Chatterjee

Academic Editor

PLOS Neglected Tropical Diseases

Hira Nakhasi

Section Editor

PLOS Neglected Tropical Diseases

Shaden Kamhawi

co-Editor-in-Chief

PLOS Neglected Tropical Diseases

orcid.org/0000-0003-4304-636XX

Paul Brindley

co-Editor-in-Chief

PLOS Neglected Tropical Diseases

orcid.org/0000-0003-1765-0002

**Additional Editor Comments (if provided):**

Several shortcomings have been assessed, if you are able to address the same. we are providing you an opportunity.

**Journal Requirements:**

If the reviewer comments include a recommendation to cite specific previously published works, please review and evaluate these publications to determine whether they are relevant and should be cited. There is no requirement to cite these works unless the editor has indicated otherwise.

1) Please ensure that the CRediT author contributions listed for every co-author are completed accurately and in full.

At this stage, the following Authors/Authors require contributions: Eyob Girma Abera. Please ensure that the full contributions of each author are acknowledged in the "Add/Edit/Remove Authors" section of our submission form.

The list of CRediT author contributions may be found here: https://journals.plos.org/plosntds/s/authorship#loc-author-contributions

2) Please provide an Author Summary. This should appear in your manuscript between the Abstract (if applicable) and the Introduction, and should be 150-200 words long. The aim should be to make your findings accessible to a wide audience that includes both scientists and non-scientists. Sample summaries can be found on our website under Submission Guidelines:

https://journals.plos.org/plosntds/s/submission-guidelines#loc-parts-of-a-submission

3) We do not publish any copyright or trademark symbols that usually accompany proprietary names, eg ©,  ®, or TM  (e.g. next to drug or reagent names). Therefore please remove all instances of trademark/copyright symbols throughout the text, including:

- ® on page: 32.

4) We have amended your Competing Interest statement to comply with journal style. We kindly ask that you double check the statement and let us know if anything is incorrect.

**Reviewers' Comments:**

Reviewer's Responses to Questions

**Key Review Criteria Required for Acceptance?**

As you describe the new analyses required for acceptance, please consider the following:

**Methods**

-Are the objectives of the study clearly articulated with a clear testable hypothesis stated?

-Is the study design appropriate to address the stated objectives?

-Is the population clearly described and appropriate for the hypothesis being tested?

-Is the sample size sufficient to ensure adequate power to address the hypothesis being tested?

-Were correct statistical analysis used to support conclusions?

-Are there concerns about ethical or regulatory requirements being met?

Reviewer #1: See general comments

Reviewer #2: Please see under Summary and General Comments

**********

**Results**

-Does the analysis presented match the analysis plan?

-Are the results clearly and completely presented?

-Are the figures (Tables, Images) of sufficient quality for clarity?

Reviewer #1: See general comments

Reviewer #2: Please see under Summary and General Comments

**********

**Conclusions**

-Are the conclusions supported by the data presented?

-Are the limitations of analysis clearly described?

-Do the authors discuss how these data can be helpful to advance our understanding of the topic under study?

-Is public health relevance addressed?

Reviewer #1: See general comments

Reviewer #2: Please see under Summary and General Comments

**********

**Editorial and Data Presentation Modifications?**

Use this section for editorial suggestions as well as relatively minor modifications of existing data that would enhance clarity. If the only modifications needed are minor and/or editorial, you may wish to recommend “Minor Revision” or “Accept”.

Reviewer #1: See general comments

Reviewer #2: (No Response)

**********

**Summary and General Comments**

Use this section to provide overall comments, discuss strengths/weaknesses of the study, novelty, significance, general execution and scholarship. You may also include additional comments for the author, including concerns about dual publication, research ethics, or publication ethics. If requesting major revision, please articulate the new experiments that are needed.

Reviewer #1: From Visceral to Visible: A Systematic Review and Meta-Analysis of Post-Kala-Azar

Dermal Leishmaniasis Incidence, Risk Factors, and Treatment Outcomes in Eastern

Africa

Major comments:

The authors conducted a systematic literature review on PKDL. In particular they looked at risk of PKDL after VL treatment, taking into account possible associated factors such as treatment regimen for VL, geographic site, age, sex. They conclude that there’s a higher risk for PKDL in East Africa, though heterogeneity within the region is substantial. Highest risk was found in Sudan, young age was also a risk factor. Treatment of primary VL episode was a key risk factor, with PKDL risk decreasing as SSG monotherapy was abandoned. They also looked at treatment of CL, MF+PM seemed very promising.

This is a well written and interesting manuscript, there are just some things that could be better clarified. In particular it is not always clear how findings are linked to the main objectives. For results on treatment it’s not always clear whether it’s about treatment of PKDL or of the original VL episode. Also at times it appears risk factors overlap, i.e. Sudan and SSG monotherapy. It would be good to disentangle these effects or explain why this was not possible.

Line 257-259: ‘Sudan emerged as the clear high-risk epicenter with an exceptional pooled PKDL cumulative incidence of 56.7%’.

Comment: Does this difference still persist after controlling for treatment regimen?

Line 346-349: ‘On a localized scale, recent field data from rural Kenya highlighted intense micro-geographical clustering, demonstrating that risk boundaries vary significantly between adjacent administrative sectors, such as Tiaty East versus Tiaty West (p=0.03) [19]’.

Comment: is this about risk for PKDL or risk for VL? If it is about PKDL, did you adjust for VL incidence?

Line 357-358 : ‘co-infected patients had an initial cure rate of only 56% compared to 95.1% overall’.

Comment: Again not clear if this is about VL or PKDL, if it is about VL, does it belong in this manuscript?

Line 446-447: ‘The between-study variance was demonstrably attributable to country-level differences, study design, and operational era rather than random sampling error.’

Comment: Was it not the treatment regimen used in the VL episode that was the most important factor (as mentioned in lines 463-469) ?

Line 491-493: ‘Sudanese longitudinal cohorts documented that infants and toddlers aged 0–3 years faced a cumulative PKDL risk of 78%–81% under historical antimonial environments [16,17].’

Comment: Is there also evidence that older age groups in this cohort that were treated with the same regimen had significantly lower risk of PKDL?

Line 507-509: ‘our review uncovered compelling evidence of micro geographical PKDL clustering, with significant incidence variation between adjacent administrative sectors in Kenya (Tiaty East vs. Tiaty West; p=0.03) [19]’.

Comment: See earlier comment, did you take into account the clustering of primary VL? If you explain it by vector ecology (line 510-511) then it is about VL clustering in the first place.

Minor comments:

Line 40: ‘heterogeneity was assessed using the I² statistic’.

Comment: Please provide a reference.

Line 158: ‘Data on incidence were extracted and pooled using random-effects meta-analysis’.

Comment: Please specify which level of random effects, study, country, both?

Line 248: ‘Among the total 924 patients, the pooled cumulative incidence of PKDL among treated visceral leishmaniasis patients in East Africa was 18.2% (95% CI: 4.7%–49.7%)’

Comment: Not clear where the 924 comes from since in the paragraph above it is stated that the total sample size was 4,699.

Line 325-328: ‘Conversely, data from the 3,126-patient prospective pharmacovigilance registry indicated that advanced age over 50 years acts as a protective factor, demonstrating significantly higher cure rates (81.4%) compared to younger patients (95.1% overall) [9]’.

Comment: What is meant here by cure rate and why is the cura rate higher for younger patients if older age is a protective factor? Why is this mentioned in the section of risk factors for PKDL?

Reviewer #2: The manuscript provides a systematic review and meta-analysis on aspects of PKDL using PRISMA 2020 guidelines and JBI methodology.

While the methodology seems sound, the results do not represent the published literature and therefore deviate from what we know on the epidemiology and pathophysiology of VL and PKDL in the region, as this was not captured in the papers examined.

For example:

1. Differences in VK in East Africa should be clear

a. From the older literature, the parasite is L. donovani but the strain in Sudan / Northern Ethiopia is different from that found in South Ethiopia / Kenya.

b. Also from the older literature, the vector is different: in Sudan /Northern Ethiopia P. orientalis, while in South Ethiopia / Kenya it is mainly P. martini.

c. However, in the recent outbreak in Kenya the L. donovani that is circulating may be yet another subtype and P. orientalis may play a more important role with higher infection rates. It is however not clear if these findings are really new or may not have been detected in previous years.

2. The genetic background of the population is important: even in the endemic area in Sudan there are differences in PKDL rates between villages that basically share the same ecology with the Masaleet as the most affected tribe. Clearly, as a whole the Sudanese and South Sudanese populations are different from those in Ethiopia and Kenya.

3. On the same note HIV co-infection in VL is common in Ethiopia while in Sudan it is rarely found.

4. The studies examined differ in design and follow-up time. For example the RCTs quoted have strict entry criteria while the clinical studies are likely to reflect the true situation. In addition, in the RCTs the follow-up time is 6 months while it is clear that PKDL in Sudan mostly occurs between 0-13 months after VL treatment.

5. The high PKDL rate that was reported from Sudan in early studies was caused by adulterated (mixed with water) SSG administered by unqualified health workers in too low dosage and intermittent duration. This suggested undertreatment as an important risk factor for PKDL. Subsequent studies with supervised treatment and proper follow-up showed much lower PKDL rates and the last available study showed 20-28%.

6. The resistance issue towards SSG should be discussed on how it differs between Asia and Africa, also with regard to PKDL incidence.

7. What is the explanation for the differences between males and females with regard to PKDL risk? Mentioning this is only relevant if you present potential explanations.

8. The issue of persisting parasites in the skin after VL treatment is not discussed as well as PK/PD issues in VL treatment.

Specific comments

Line 61: not only L. donovani

Line 76: ref 7 may not be the correct one

Line 131-4: this is probably the problem in the methodology

Line 304: disease velocity ?

Line 337-351: this needs much better analysis and discussion

Line 444: this needs to be discussed in detail. The ref seems not correct.

Line 462 and 469: there should be better refs for this than Proceedings of this meeting

Line 492: what are historical antimonial environments? Please avoid phrases or concepts that are not common language.

**********

PLOS authors have the option to publish the peer review history of their article (what does this mean?). If published, this will include your full peer review and any attached files.

If you choose “no”, your identity will remain anonymous but your review may still be made public.

**Do you want your identity to be public for this peer review?**  For information about this choice, including consent withdrawal, please see our Privacy Policy.

Reviewer #1: No

Reviewer #2: No

[NOTE: If reviewer comments were submitted as an attachment file, they will be attached to this email and accessible via the submission site. Please log into your account, locate the manuscript record, and check for the action link "View Attachments". If this link does not appear, there are no attachment files.]

**Figure resubmission:**

**Reproducibility:**

To enhance the reproducibility of your results, we recommend that authors of applicable studies deposit laboratory protocols in protocols.io, where a protocol can be assigned its own identifier (DOI) such that it can be cited independently in the future. Additionally, PLOS ONE offers an option to publish peer-reviewed clinical study protocols. Read more information on sharing protocols at https://plos.org/protocols?utm_medium=editorial-email&utm_source=authorletters&utm_campaign=protocols

# Author comment: Author response to Decision Letter 1

**DOI:** 10.1371/journal.pntd.0014666.r002

23 Jul 2026

Attachment Submitted filename: Response to Reviewers.docx

# Editor report: Decision Letter 1

**DOI:** 10.1371/journal.pntd.0014666.r003

**Authors:** Hira Nakhasi

10 Aug 2026

Dear Mr Abera,

We are pleased to inform you that your manuscript 'From Visceral to Visible: A Systematic Review and Meta-Analysis of Post-Kala-Azar Dermal Leishmaniasis Incidence, Risk Factors, and Treatment Outcomes in Eastern Africa' has been provisionally accepted for publication in PLOS Neglected Tropical Diseases.

Before your manuscript can be formally accepted you will need to complete some formatting changes, which you will receive in a follow up email. A member of our team will be in touch with a set of requests.

Please note that your manuscript will not be scheduled for publication until you have made the required changes, so a swift response is appreciated.

IMPORTANT: The editorial review process is now complete. PLOS will only permit corrections to spelling, formatting or significant scientific errors from this point onwards. Requests for major changes, or any which affect the scientific understanding of your work, will cause delays to the publication date of your manuscript.

Should you, your institution's press office or the journal office choose to press release your paper, you will automatically be opted out of early publication. We ask that you notify us now if you or your institution is planning to press release the article. All press must be co-ordinated with PLOS.

Thank you again for supporting Open Access publishing; we are looking forward to publishing your work in PLOS Neglected Tropical Diseases.

Best regards,

Hira L Nakhasi, Ph.D.

Section Editor

PLOS Neglected Tropical Diseases

Hira Nakhasi

Section Editor

PLOS Neglected Tropical Diseases

Shaden Kamhawi

co-Editor-in-Chief

PLOS Neglected Tropical Diseases

orcid.org/0000-0003-4304-636XX

Paul Brindley

co-Editor-in-Chief

PLOS Neglected Tropical Diseases

orcid.org/0000-0003-1765-0002

***********************************************************

The authors have revised the paper as per reviewers comments.

p.p1 {margin: 0.0px 0.0px 0.0px 0.0px; line-height: 16.0px; font: 14.0px Arial; color: #323333; -webkit-text-stroke: #323333}span.s1 {font-kerning: none

# Editor report: Acceptance letter

**DOI:** 10.1371/journal.pntd.0014666.r004

**Authors:** Hira Nakhasi

Dear Mr Abera,

We are delighted to inform you that your manuscript, "From Visceral to Visible: A Systematic Review and Meta-Analysis of Post-Kala-Azar Dermal Leishmaniasis Incidence, Risk Factors, and Treatment Outcomes in Eastern Africa," has been formally accepted for publication in PLOS Neglected Tropical Diseases.

We have now passed your article onto the PLOS Production Department who will complete the rest of the publication process. All authors will receive a confirmation email upon publication.

The corresponding author will soon be receiving a typeset proof for review, to ensure errors have not been introduced during production. Please review the PDF proof of your manuscript carefully, as this is the last chance to correct any scientific or type-setting errors. Please note that major changes, or those which affect the scientific understanding of the work, will likely cause delays to the publication date of your manuscript. Note: Proofs for Front Matter articles (Editorial, Viewpoint, Symposium, Review, etc...) are generated on a different schedule and may not be made available as quickly.

Soon after your final files are uploaded, the early version of your manuscript will be published online unless you opted out of this process. The date of the early version will be your article's publication date. The final article will be published to the same URL, and all versions of the paper will be accessible to readers.

For Research Articles, you will receive an invoice from PLOS for your publication fee after your manuscript has reached the completed accept phase. If you receive an email requesting payment before acceptance or for any other service, this may be a phishing scheme. Learn how to identify phishing emails and protect your accounts at https://explore.plos.org/phishing.

Thank you again for supporting open-access publishing; we are looking forward to publishing your work in PLOS Neglected Tropical Diseases.

Best regards,

Shaden Kamhawi

co-Editor-in-Chief

PLOS Neglected Tropical Diseases

Paul Brindley

co-Editor-in-Chief

PLOS Neglected Tropical Diseases
